# Nationwide Subjective and Objective Assessments of Potential Talent Predictors in Elite Youth Soccer: An Investigation of Prognostic Validity in a Prospective Study

**DOI:** 10.3389/fspor.2021.638227

**Published:** 2021-05-28

**Authors:** Oliver Höner, Dennis Murr, Paul Larkin, Robert Schreiner, Daniel Leyhr

**Affiliations:** ^1^Institute of Sports Science, Eberhard Karls University of Tübingen, Tübingen, Germany; ^2^Institute for Health and Sport, Victoria University, Melbourne, VIC, Australia; ^3^Methods Center, Eberhard Karls University of Tübingen, Tübingen, Germany

**Keywords:** football, coach's eye, talent identification and development, tactical skills, technical skills, physiological abilities, psychosocial skills, multidimensional diagnostic

## Abstract

Recent studies have provided empirical evidence on the prognostic relevance of objective performance diagnostics in the soccer talent identification and development process. However, little is known about the prognostic validity of coaches' subjective evaluations of performance. This study evaluated objective and subjective assessments within a nationwide talent development program and addressed motor, perceptual skill, and personality-related performance factors. Male players (*N* = 13,869; *M*_*age*_ = 12.59 ± 1.07 years) from the age groups U12 to U15 of the German soccer talent development program participated in this study. Participants completed an objective motor diagnostic (sprint, agility, dribbling, ball control, juggling) and were subjectively rated by their coaches (kicking skills, endurance, individual tactical skills, psychosocial skills). All nine predictors were assessed with sufficient psychometric properties (α ≥ 0.72; except dribbling and ball control: α ≥ 0.53). Players' success three seasons later was operationalized by achieving professional youth academy level or not (success rate, 9%). Independent-samples *t*-tests analyzed univariate mean group comparisons between future selected and non-selected players. Logistic regression models examined the multivariate prognostic validity of all assessments by predicting success with subjective (model 1), objective (model 2), and both groups of predictors (model 3). Confirming the univariate prognostic validity, future selected outperformed non-selected players regarding all predictors (each *p* < 0.001, except for agility in U15: *p* < 0.01). Tactical skills, kicking skills, and sprint were of highest predictive value (*d* ≥ 0.61 in each age group). Multivariate results provided empirical evidence for the subjective (7% ≤ Nagelkerke's *R*^2^ ≤ 11%; each *p* < 0.001) and objective (8% ≤ Nagelkerke's *R*^2^ ≤ 13%; each *p* < 0.001) assessments' prognostic validity. However, model 3 revealed the best statistical explanatory power in each age group (0.15 ≤ Nagelkerke's *R*^2^ ≤ 0.20; *p* < 0.001). In this combined assessment model, sprint, tactical skills, and dribbling were found to be the most predictive variables. In conclusion, this study reinforces the call for multidimensional diagnostics integrating objective and subjective assessments. Future research is needed to address the demands for longitudinal analyses of subjective ratings, the integration of biological maturation, and empirical evidence for female soccer.

## Introduction

Talent identification and development in soccer have been “vibrant research areas” for sport scientists in the last two decades (Williams et al., 2020, p. 1). Several prospective studies with multidimensional approaches have provided empirical evidence on the significant, yet also partly limited prognostic relevance of objective diagnostics that assess youth players' characteristics, abilities, and skills in soccer (Murr et al., 2018a,b; Sarmento et al., 2018; Ivarsson et al., 2020). However, little is known about the prognostic validity of subjective evaluations of such performance factors by coaches or scouts, and recent studies highlight the need to integrate both subjective and objective evaluations of potential talent predictors (Dugdale et al., 2020; Ford et al., 2020).

The identification of talent in soccer has been studied from a variety of theoretical and methodological approaches (Williams et al., 2020). Since soccer is a complex team sport, a number of performance factors must be considered when determining which youth athletes have the highest potential to develop into elite players (Lund and Söderström, 2017). For example, researchers have highlighted different qualities associated with performance, including physiological, technical, tactical, and psychological attributes (Hoare and Warr, 2000; Unnithan et al., 2012; Suppiah et al., 2015). With respect to physiological performance measurements, researchers indicate that selected youth soccer players are faster than non-selected players (Gil et al., 2014; Höner and Votteler, 2016). Furthermore, researchers found players who progressed to an elite level were more technically competent for skills such as juggling, dribbling, and passing accuracy (Höner et al., 2017; Bergkamp et al., 2019). In addition, skilled players have been found to possess superior perceptual–cognitive skills when compared to less skilled counterparts (Ward et al., 2013; O'Connor et al., 2016). Finally, psychological attributes such as self-confidence, motivation, mental toughness, commitment, and seeking social support have also been found to predict elite level soccer career success (Williams and Reilly, 2000; Toering et al., 2009; Van Yperen, 2009; Baláková et al., 2015; Höner and Feichtinger, 2016). Most of these findings were based on objective measurements and provide an indication of the factors that may predict future high performance. However, due to the considerable variation in study designs, findings across individual talent identification studies are inconsistent and difficult to compare. Therefore, there is no clear set of variables that uniformly predicts skill level (Breitbach et al., 2014; Höner and Feichtinger, 2016; Bergkamp et al., 2018; Johnston et al., 2018; Murr et al., 2018b).

Given the challenges associated with talent identification, standard talent identification procedures often rely on the evaluation of athletes' current performance by coaches and scouts (Williams et al., 2020). Within this process, coaches' experience and expertise in identifying potential talent comprise an important tool (Larkin et al., 2020). To this end, researchers have used interview techniques to determine what experienced soccer coaches look for when identifying potential talent. Christensen (2009) found Danish national team coaches valued game intelligence (i.e., ability to read and predict game play) and soccer-specific physiological and technical skills as the most important factors when assessing talent. Coaches also considered personal qualities (e.g., character, attitude, drive to succeed, and willingness to learn) as important. This finding is further supported by Larkin and O'Connor (2017), who identified a hierarchy of attributes perceived as important by coaches when identifying youth soccer players: technical (i.e., first touch, kicking skills, one-vs.-one ability, technical ability under pressure), tactical (i.e., decision-making skills), and psychological attributes (i.e., coachability, positive attitude).

Specifically related to the process of talent selection, Lund and Söderström (2017) found that Swedish soccer coaches made decisions based on previous experience, current elite players' qualities, and the values and belief system of the club. This suggests the decision to select or not to select an athlete is based on intuition and deliberation, grounded on an “overall impression,” factoring in subjective evaluations of technical skills, game understanding, and a variety of psychological characteristics (Meylan et al., 2010; MacMahon et al., 2018; Williams et al., 2020). However, based on knowledge from selection psychology, it is argued that individuals should be cautious when using such “clinical judgments” (Dawes et al., 1989), as various pieces of information have to be combined to make a decision, and as such, there is potential for different errors and biases. As a result, this may lead to less accurate decisions and inconsistencies between individual decision-makers (Den Hartigh et al., 2018). This issue has been identified with evidence to suggest the accuracy of subjective talent decisions by coaches and scouts is relatively low. Koz et al. (2011) found the accuracy of selection decisions for professional sports “entry drafts” suggests that even when these decisions are made late in development (i.e., early adulthood), the level of predictive accuracy is comparatively low.

Therefore, Ford et al. (2020) suggested researchers consider the integration of both subjective and objective evaluations of performance factors. However, according to the review of Williams et al. (2020), only two studies have explored the relationship between subjective evaluations and objective tests in this context. In a prospective design, Sieghartsleitner et al. (2019a) examined the isolated and combined prognostic relevance of several characteristics for the identification of future playing status, including the subjective evaluation of players' in-game performance in addition to objective diagnostics of technical and general motor performance factors. Results indicated that the use of subjective coach assessments and objective performance data was significantly better at predicting under 19 (U19) player status than objective performance data alone. More recently, Dugdale et al. (2020) compared levels of agreement between subjective and objective assessment of youth elite Scottish soccer players' (U11–U17 age group) physical performance. Athletes completed different physical performance assessments (e.g., endurance, acceleration, speed), with coaches providing a subjective evaluation of the physical variables. The findings indicated the coaches' subjective evaluation only corresponded with high and low performance on the objective physical assessments, suggesting that coaches' subjective evaluations may not be sensitive enough to discriminate between players whose performance level is rather similar. In conclusion, the two studies highlight the importance of combining both subjective evaluations and objective test results within the talent identification process. It should be noted, however, that Sieghartsleitner et al. (2019a) did not examine subjective performance factors, but general in-game performance, and Dugdale et al. (2020) did not address the prognostic validity of subjective rated performance factors with regards to players' future performance level. Thus, given the lack of data on coaches' and scouts' efficacy regarding selection decisions, there is a need to examine the prognostic validity of subjective and objective evaluations of youth players' performance factors in regard to their validity for talent selections over an extended period.

## The Present Study

With an applied focus on the key process of talent selection, the present study was conducted within the talent development program of the German Soccer Association (Deutscher Fußball-Bund, DFB). In this program, two nationwide assessments of potential talent predictors are implemented to monitor the development of players' performance factors. First, a motor test battery was implemented as an objective diagnostic in 2004 and is conducted semiannually. Second, starting with the 2015/2016 season, coaches involved in the program rate players on subjective evaluation criteria in the spring of each season. This subjective evaluation supplements the objective motor diagnostic that currently addresses technical skills (i.e., ball control, dribbling, juggling) as well as linear and change of direction speed abilities (i.e., sprinting, agility^1^).

Given these two existing nationwide assessments in the German talent development program, the aim of this prospective study was to investigate the objective diagnostic and subjective evaluation in the age groups U12–U15 in relation to their prognostic validity for future success. First, within a *univariate perspective*, we examined the prognostic validity of each single performance factor that was measured with an objective or subjective assessment. Second, within a *multivariate perspective*, we evaluated whether the (objective, subjective, and combined) assessments as a whole provide a meaningful prognostic model for players' future success and compared the contributions of each performance factor within these assessments.

## Methods

### Sample and Design

Within the German talent development program, nearly 1,300 part-time DFB coaches select about 14,000 early-adolescent players (i.e., age groups U12–U15) from amateur clubs all over the country to participate in one additional practice session per week at one of the 366 regional competence centers (CC). This quite homogeneous group of high-performing youth players belong to the top 4% of all German male youth players in their age group (Deutscher Fußball Bund, 2009). A central purpose of this program is the development of these CC players with the aim that they will be selected for one of the professional German soccer clubs' youth academies in middle-to-late adolescence (YA, top 1%).

The sample of this *prospective cohort study* comprised *N* = 13,869 male CC players (*M*_*age*_ = 12.59 ± 1.07 years; age groups U12–U15; birth cohorts: 2001–2005) who participated in the nationwide motor diagnostic (Höner et al., 2015). Furthermore, players were subjectively rated by their coaches regarding several performance factors either in spring of the 2015/2016 season (birth cohorts 2001–2004) or in spring of the 2016/2017 season (birth cohorts 2002–2005). The objectively and subjectively evaluated performance factors served as predictors for *players' future success* three seasons later.

Before entering the talent development program, players' legal guardian provided written informed consent for the recording and scientific use of the data. The CC coaches together with DFB staff members conducted the motor diagnostic and performed the subjective evaluations of the present study. The DFB provided the authors with players' data of the five birth cohorts (2001–2005). The university's ethics department approved the implementation of this study.

### Measures

#### Predictors

The *subjective performance evaluation* was conducted by the 1,300 coaches of the German talent development program who possess at least the UEFA B-License. Coaches' subjective evaluations of motor (i.e., kicking skills, endurance), perceptual–cognitive (i.e., individual tactical skills), and personality-related domain (i.e., psychosocial skills) were assessed using a questionnaire consisting of 14 items (see Table 1). With respect to the motor domain, three items assessed kicking skills (i.e., kicking the ball with the dominant and non-dominant leg, heading the ball), and one item addressed endurance as a physiological performance factor. Regarding the perceptual–cognitive domain, individual tactical skills were subjectively assessed by the quality of individual tactical behavior in offensive and defensive situations (i.e., before, during, and after ball-related actions) as well as overall game intelligence (i.e., seven items in total). Finally, within the personality-related domain, the assessment of psychosocial skills comprised three items including motivational, volitional, and social skills (one item each). To enhance a nationwide common understanding of the items, the CC coaches were educated by DFB staff members, and a 16-page manual was distributed to the coaches before implementing the subjective evaluation. For each item, the manual included key points as well as detailed explanations and examples. Both the key points and examples guided the CC coaches what to look for in their subjective ratings.

**Table 1:** Subjectively assessed youth players' performance factors serving as potential predictors for future success.

**Domain**	**Performance factor (# items)**	**Items for subjective evaluation of youth players' performance factors**
Motor	Kicking skills (3)	– Kicking the ball with ◦ dominant leg ◦ non-dominant leg – Heading
	Endurance ability (1)	– Endurance
Perceptual–cognitive	Individual tactical skills (7)	– Behavior in offensive situations ◦ before ball-related actions ◦ during ball-related actions ◦ after ball-related actions – Behavior in defensive situations ◦ before ball-related actions ◦ during ball-related actions ◦ after ball-related actions – Game intelligence
Personality related	Psychosocial skills (3)	– Motivational skills – Volitional skills – Social skills

Table 2 provides an extract of the manual in regard to the three items addressing individual tactical behavior in offensive situations. For the item “individual tactical behavior in offensive situations *before* ball-related actions,” the CC coaches were directed by the manual to focus their subjective evaluation on the key points “preorientation” and “offering/creating space.” To get a more vivid and concrete understanding, the two key points were explained by typical examples: (1) CC players can orientate themselves in such a way that they make an appropriate decision (e.g., find an open position, look over their shoulders), and (2) CC players can make themselves available (e.g., separate themselves from a defender) in such a way that they can receive a pass or create space in which another players can receive a pass. The individual tactical behavior in offensive situations *during* ball-related actions was concretized by the key points “first touch”, “orientation on the ball”, and “situation-appropriate decision-making”, and regarding the behavior in offensive situations *after* ball-related actions, coaches should pay attention to the “reorientation” and “follow-up action” (these key points were also illustrated by typical examples, see Table 2; for corresponding information regarding the other performance factors, see Supplementary Table 1).

**Table 2:** Key points and their explanations for rating players' individual tactical skills in offensive situations (before, during, and after ball-related actions).

***Behavior in offensive situations…***	**Key points**	**Explanation of the key points**
**Before ball-related actions**	➢ Preorientation ➢ Offering/creating space	Competence center players can… • Orientate themselves in such a way that they make an appropriate decision: e.g., open, look over their shoulders. • Make themselves available in such a way that they are playable or create space in which another player becomes playable: e.g., separate themselves from a defender.
**During ball-related actions**	➢ First touch ➢ Orientation on the ball ➢ Situation-appropriate decision-making	Competence center players can… • Play the ball with the first touch according to the situation: e.g., in the new direction of play, in the open space/away from the opponent, secure the ball. • Orient themselves while they are on the ball: e.g., glance away from the ball, view the next situation. • Decide appropriately: e.g., pass the ball to a teammate, dribble with the ball, shoot the ball toward the goal.
**After ball-related actions**	➢ Re-orientation ➢ Follow-up action	Competence center players can… • Act according to a new situation after a play: e.g., pass the ball and immediately find an opening again, be offset to back up teammates, be open again, create space for teammates.

Coaches were asked to judge each item from a holistic perspective (i.e., for their “overall impression” about the player in the respective season). For the motor and perceptual–cognitive performance factors, coaches rated their CC players' performance in comparison to the general CC player level and to the level of regional association team players (i.e., the next highest level within the talent development program). These reference levels were familiar to all CC coaches and thus implemented for the subjective assessment to ensure that CC coaches not only had a similar idea of the items (see Table 2) but also a reference norm that was as consistent as possible regarding the performance level. Thus, for each item, coaches evaluated their players on a 4-point rating scale as “below-average DFB competence center level” (0); “average DFB competence center level” (1); “level of the extended squad for regional association team” (2); or “level of core team for the regional association team” (3). As it was difficult for CC coaches to judge the psychosocial skills on these levels, no direct relation to the general CC or regional association team level was established. Thus, for the evaluation of the psychosocial skills items, the coaches rated their players as “below average level” (0), “average level” (1), “high level” (2), or “very high level” (3). Based on these ratings, a value for each respective performance factor was calculated by the average of the corresponding individual items (e.g., the mean of the three judgements for the kicking skills items represented the indicator for the subjective evaluation of the player's kicking skills).

The *objective motor diagnostic* included five individual tests to assess players' speed abilities and technical skills (for details, see Höner et al., 2015): sprint (i.e., time in a 20 m linear sprint); agility (i.e., time in a slalom course without a ball); dribbling (time in a slalom course with a ball); ball control (i.e., time needed to play six passes alternately against two opposing impact walls with at least two ball contacts); and ball juggling (i.e., juggle the ball alternately with the left and right foot through as many subsections of a figure eight-course without ground contact). Light barrier systems (Brower TC Timing, Draper, USA) were utilized to measure execution times for sprint, agility, and dribbling. Times for ball control were assessed by hand-stopped chronographs. Each test was performed twice with the best result recorded for analysis purposes. Between the attempts, the athletes were given enough time to recover. Whereas the time-based individual tests (i.e., sprint, agility, dribbling, and ball control) were negatively coded (i.e., a lower value indicated a better performance), a higher value in the juggling test represented higher performance.

As the nationwide assessments were more relevant for comparisons of players within age groups than over several age groups, the *psychometric properties* for the predictors in this study were investigated for each age group separately. The subjective performance scales were characterized by excellent reliability values in terms of internal consistency for tactical skills (0.89 ≤ α ≤ 0.91 for U12, U13, U14, and U15) and psychosocial skills (0.84 ≤ α ≤ 0.87), whereas kicking skills (0.73 ≤ α ≤ 0.77) showed at least satisfying values. Moreover, Höner et al. (2015) analyzed the age-specific motor test battery's psychometric properties for a sample of nearly 70,000 male CC players and found excellent internal consistency for sprint (0.92 ≤ α ≤ 0.93 for U12, U13, U14, and U15) and agility (0.90 ≤ α ≤ 0.90). Juggling revealed satisfying values (0.72 ≤ α ≤ 0.75), whereas those for dribbling (0.53 ≤ α ≤ 0.57) and ball control (0.61 ≤ α ≤ 0.64) were slightly lower.

#### Criterion

Three seasons after participating at the nationwide assessment in the competence centers (i.e., time of predictor data collection), players' future success was operationalized by achieving the German YA level or not (i.e., *selected* vs. *non-selected*). In general, the CC coaches who conducted the objective and subjective assessments were not involved in the future selection process for the YAs. Moreover, the approach ensured the same prognostic period (i.e., three seasons) for all players. The selected group comprised players who participated at the assessments in spring 2015 and were enrolled in a YA for the 2018/2019 season and those who completed the tests in spring 2016 and were enrolled in a YA for the 2019/2020 season. To identify the enrolled YA players, the squad lists for the respective age groups of all German YA were examined regarding the players' names and birth dates. Players who were—according to the squad lists in the respective seasons—identified as YA players were defined as *selected* and the others as *non-selected* players. Overall, *n* = 1,198 players were categorized as selected and *n* = 12,671 players as non-selected players (i.e., the success rate for achieving the youth academy level in this study was about 9%).

### Statistical Analysis

Data were analyzed utilizing IBM SPSS version 26. To provide robust results regarding the predictors' prognostic relevance, the data from the two measurement points (i.e., season 2015/2016 and 2016/2017) was accumulated for each age groups (U12–U15). If a player participated in the assessment in both seasons, only the data from the first assessment was taken. However, the two seasons and the corresponding birth cohorts in the sample (2001–2004 and 2002–2005, respectively) may confound the analysis of performances' differences between selection levels (Elferink-Gemser et al., 2012). To control for this assumption, two-way ANOVAs for each performance factor were conducted testing whether there was an interaction effect between future success and the respective birth cohorts tested at the first and second season. As non-significant interactions [*F*_(1,13865)_ ≤ 3.36, *p* ≥ 0.07] were found for all variables, the cohort variable was not considered as a confounder in the following analysis. Similarly, the influence of relative age as a potential confounder within the analysis was investigated, and non-significant interactions [*F*_(1,13865)_ ≤ 3.12, *p* ≥ 0.08] between future success and relative age (player born in first or second half of the year) were found.

As coaches' subjective evaluations were ratings in relation to the respective age group (i.e., U12, U13, U14, and U15), the following statistical analyses were conducted for each age group separately. The variance of the investigated variables within each age group of the preselected samples and, accordingly, the expected statistical effect sizes may be limited in talent research studies (restriction of range of talent; Ackerman, 2014). Moreover, single predictors may represent only a small part of complex soccer performance. Thus, test power or sensitivity in prospective talent studies is a critical issue (Bergkamp et al., 2019). To determine the size of a possibly detected population effect for differences between two groups, sensitivity was calculated by *post-hoc* power analyses using G^*^Power version 3.1.9.7 and predetermined parameters (α = 0.05, 1 – β = 0.85, two-tailed). For the age groups U12, U13, U14, and U15 and their corresponding sample sizes (see Table 3), the analyses were sensitive enough to detect small to medium effect sizes *d* ≥ 0.13, *d* ≥ 0.17, *d* ≥ 0.22, and *d* ≥ 0.40, respectively.

**Table 3:** Test results for subjectively (printed in italics) and objectively evaluated performance factors of selected and non-selected players separated by age class (U12–U15, *N* = 13,869).

**Performance factor**	**Future performance level (after 3 seasons)**	***M*** **±** ***SD***				***t (df)* Cohen's *d*^+^ (95% CI)**			
		**U12**	**U13**	**U14**	**U15**	**U12**	**U13**	**U14**	**U15**
	Selected (*N*)	582	356	202	58				
	Non-selected (*N*)	6,077	3,324	2,083	1,187				
*Kicking skills*	Selected	1.71 ± 0.53	1.76 ± 0.55	1.70 ± 0.51	1.88 ± 0.42	15.72 (6,657)	15.12 (5,703)	10.17 (3,454)	6.80 (1,963)
	Non-selected	1.33 ± 0.56	1.37 ± 0.54	1.38 ± 0.56	1.48 ± 0.54	0.70^***^ (0.60; 0.77)	0.72^***^ (0.61; 0.83)	0.61^***^ (0.43; 0.72)	0.82^***^ (0.48; 1.01)
*Endurance*	Selected	2.05 ± 0.72	2.03 ± 0.74	2.03 ± 0.67	2.19 ± 0.61	10.27 (704.86)	8.13 (699.57)	7.59 (392.07)	3.99 (1,963)
	Non-selected	1.70 ± 0.75	1.75 ± 0.75	1.77 ± 0.73	1.90 ± 0.73	0.48^***^ (0.38; 0.55)	0.37^***^ (0.26; 0.48)	0.43^***^ (0.21; 0.50)	0.43^***^ (0.14; 0.66)
*Tactical skills*	Selected	1.89 ± 0.57	1.90 ± 0.57	1.84 ± 0.55	2.05 ± 0.51	17.26 (6,657)	14.45 (5,703)	11.32 (3,454)	7.36 (1,963)
	Non-selected	1.46 ± 0.58	1.48 ± 0.57	1.49 ± 0.57	1.59 ± 0.56	0.76^***^ (0.66; 0.83)	0.73^***^ (0.63; 0.85)	0.61^***^ (0.47; 0.76)	0.85^***^ (0.56; 1.09)
*Psychosocial skills*	Selected	2.01 ± 0.67	2.04 ± 0.64	1.99 ± 0.65	2.02 ± 0.54	10.53 (695.75)	10.51 (693.16)	6.71 (391.13)	4.40 (110.19)
	Non-selected	1.71 ± 0.66	1.72 ± 0.66	1.78 ± 0.68	1.79 ± 0.65	0.46^***^ (0.40; 0.54)	0.50^***^ (0.38; 0.60)	0.38^***^ (0.17; 0.46)	0.39^***^ (0.09; 0.62)
Sprint (20 m)^#^	Selected	3.54 ± 0.15	3.43 ± 0.15	3.29 ± 0.15	3.23 ± 0.14	14.79 (6,657)	17.64 (719.59)	12.07 (3,454)	4.98 (110.75)
	Non-selected	3.64 ± 0.16	3.55 ± 0.16	3.42 ± 0.17	3.29 ± 0.16	0.65^***^ (0.54; 0.71)	0.73^***^ (0.64; 0.87)	0.73^***^ (0.63; 0.92)	0.40^***^ (0.11; 0.64)
Agility (CODS)^#^	Selected	8.23 ± 0.42	8.14 ± 0.39	8.02 ± 0.37	7.94 ± 0.43	8.41 (6,657)	5.78 (5,703)	5.41 (3,454)	3.05 (1,963)
	Non-selected	8.38 ± 0.40	8.24 ± 0.39	8.13 ± 0.40	8.06 ± 0.39	0.36^***^ (0.29; 0.46)	0.25^***^ (0.15; 0.37)	0.29^***^ (0.13; 0.42)	0.30^**^ (0.04; 0.57)
Dribbling	Selected	10.69 ± 0.69	10.49 ± 0.65	10.30 ± 0.57	10.10 ± 0.65	11.55 (6,657)	8.78 (5,703)	8.51 (406.06)	4.63 (1,963)
	Non-selected	11.06 ± 0.74	10.77 ± 0.68	10.56 ± 0.64	10.47 ± 0.72	0.52^***^ (0.42; 0.59)	0.41^***^ (0.30; 0.52)	0.42^***^ (0.27; 0.56)	0.54^***^ (0.52; 0.64)
Ball control^#^	Selected	9.74 ± 1.25	9.37 ± 1.20	8.98 ± 1.04	8.61 ± 1.21	9.10 (6,657)	6.89 (5,703)	4.79 (3,454)	3.41 (1,963)
	Non-selected	10.27 ± 1.33	9.73 ± 1.26	9.28 ± 1.14	9.06 ± 1.17	0.41^***^ (0.32; 0.49)	0.29^***^ (0.18; 0.40)	0.22^***^ (0.12; 0.41)	0.38^**^ (0.12; 0.65)
Juggling	Selected	4.12 ± 4.24	6.13 ± 5.29	7.90 ± 5.48	9.76 ± 6.40	9.05 (638.52)	7.90 (656.82)	5.78 (372.97)	3.91 (1,963)
	Non-selected	2.50 ± 3.01	4.06 ± 4.31	6.15 ± 5.32	7.29 ± 5.74	0.52^***^ (0.43; 0.60)	0.43^***^ (0.36; 0.58)	0.35^***^ (0.18; 0.47)	0.41^***^ (0.16; 0.69)

# *These variables are negatively coded, i.e., a lower value represents a higher performance*.

+ *A positive d value displays a better test result for the selected players regardless whether the considered variable was negatively or positively coded*.

^*^p < 0.05;

** *p < 0.01*;

*** *p < 0.001*.

With respect to the *univariate prognostic validity* of the subjective evaluations as well as motor diagnostics of players' performance factors, mean group comparisons between future selected and non-selected players were computed for all assessed predictors. To identify significant differences between future successful and less-successful players, two independent samples *t*-tests were computed. Cohen's *d* (computed as the mean difference divided by the pooled standard deviation) served as effect size (including the respective 95% confidence intervals) and was classified into the categories small (0.2 ≤ *d* < 0.5), medium (0.5 ≤ *d* < 0.8), and large (*d* ≥ 0.8) in accordance with Cohen (1992). Regardless of whether the considered variable was negatively or positively coded, the provided *d* values were set positive when the selected players achieved better test results^2^.

Second, a logistic regression approach was chosen to investigate the *multivariate prognostic validity* of the assessed performance factors. Within three different logistic regression models, the binary criterion variable (selected vs. non-selected for YA) was predicted by the subjectively (*model 1*), objectively (*model 2*), and the combination of subjectively and objectively (*model 3*) assessed performance factors. Independent variables for *model 1* comprised the four subjectively judged performance factors (see Table 1), whereas *model 2* consisted of the five single motor tests. Model 3 included both the four subjective and five objective performance factors. To evaluate the whole (subjective, objective, and combined) nationwide conducted assessments, the enter method was used for each regression analysis, and each overall model fit was analyzed with the likelihood ratio chi-squared test and Nagelkerke's *R*^2^. Regression coefficients and the odds ratio coefficients *e*^*b*^ (including their 95% confidence intervals) were calculated to provide a clearer view of a player's relative chances to get selected, depending on the considered predictors. To facilitate comparisons for effect sizes of individual predictors, the odds ratio coefficients *e*^*b*^ were additionally adjusted to the standard deviations of the respective age group (Höner and Votteler, 2016). Thus, the resulting *(e*^*b*^*)*^*SD*^ represents the relative change of the likelihood for being selected for a YA by a one standard deviation increase within the considered predictor. Thereby, the adjusted odds ratios were inverted and displayed as *(e*^*b*^*)*^−*SD*^ for negatively coded predictors (i.e., the time-based tests sprint, agility, dribbling, and ball control) where lower values in time represent higher performance.

Regarding the chosen predictors within the models, considerable bivariate correlations were detected. Correlations among the four subjective performance factors in the respective age groups ranged from *r* = 0.51 (kicking skills with endurance in U15) to *r* = 0.79 (kicking skills with tactical skills in U12), while those for the five objective motor diagnostics ranged from *r* = 0.02 (sprint with juggling in U14) to *r* = 0.51 (agility with dribbling in U15). For the bivariate comparisons between subjective and objective performance factors, the highest relationship was found for endurance with sprint in U12 and U15 (i.e., each *r* = 0.22). Consequently, in order to control for a potential bias due to multicollinearity, the variance inflation factor (*VIF*) for the predictors within each logistic regression model was investigated. The *VIF* values for the predictors within the three models were all *VIF* ≤ 3.81, and because *VIFs* exceeding 10 are considered as an indicator for serious multicollinearity (Akinwande et al., 2015), no meaningful multicollinearity bias was expected for this study.

## Results

### Univariate Prognostic Validity of Subjective and Objective Performance Factors

Table 3 provides the results for the assessed predictors in the considered age groups separated by level of future success. Overall, the results for all predictors correspond to the later achieved performance level. That is, players who were selected for a YA after three seasons showed significantly superior performance factors compared to non-selected players (each *p* < 0.001, except for agility in the age group U15: *p* < 0.01).

**Figure 1:**
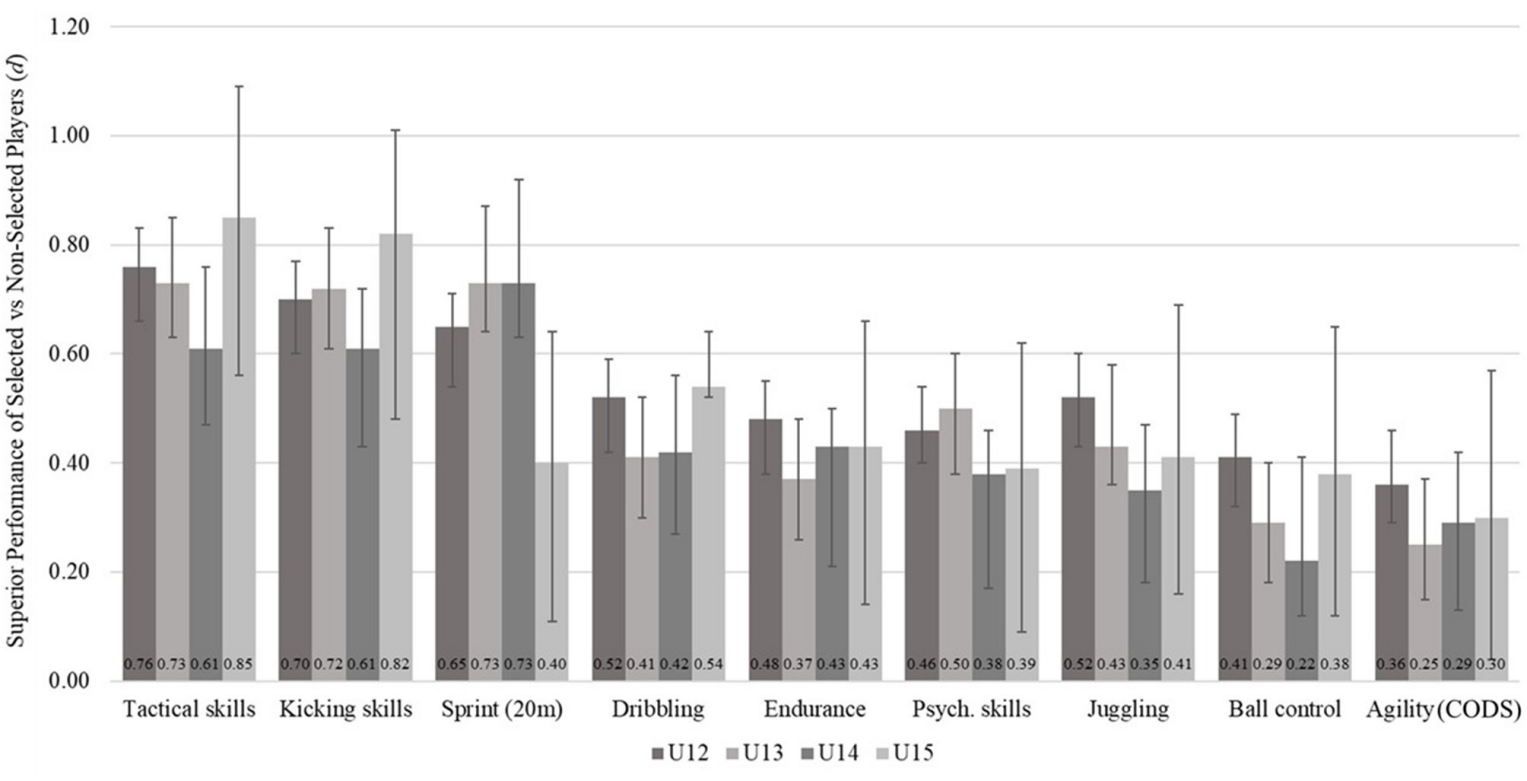
Superior performance of selected vs. non-selected players in terms of Cohen's *d* and respective 95% CI (the variables are ordered regarding the median of the effect sizes for the four age groups).

Figure 1 illustrates the superior performances of the selected compared to the non-selected players in terms of Cohen's *d*. The results indicated no clear trend for a decline or incline of the effect sizes over the age groups, that is from U12 to U15. According to the median of the effect sizes, tactical skills [*Mdn (d)* = 0.75; 0.61 ≤ *d* ≤ 0.85 for U12, U13, U14, and U15]; kicking skills [*Mdn (d)* = 0.71; 0.61 ≤ *d* ≤ 0.82]; and sprint [*Mdn (d)* = 0.69; 0.40 ≤ *d* ≤ 0.73] proved the highest discriminative power between selected and non-selected players. Dribbling [*Mdn (d)* = 0.47; 0.41 ≤ *d* ≤ 0.54]; endurance [*Mdn (d)* = 0.43; 0.37 ≤ *d* ≤ 0.48]; psychosocial skills [*Mdn (d)* = 0.43; 0.38 ≤ *d* ≤ 0.50]; and juggling [*Mdn (d)* = 0.42; 0.35 ≤ *d* ≤ 0.52] revealed nearly medium effect sizes, whereas small effect sizes were found for ball control [*Mdn (d)* = 0.34; 0.22 ≤ *d* ≤ 0.41] and agility [*Mdn (d)* = 0.30; 0.25 ≤ *d* ≤ 0.36].

Overall, the predictive power of the four subjectively rated performance factors [*Mdn (d)* = 0.56; 0.38 ≤ *d* ≤ 0.85 for all subjective predictors over all age groups] were detected to be higher than those of the objectively assessed motor performance factors [*Mdn (d)* = 0.41; 0.22 ≤ *d* ≤ 0.73]. Except for sprinting, effect sizes for the objective diagnostics revealed slightly lower values ranging from *d* = 0.22 for ball control in U14 to *d* = 0.54 for dribbling in U15.

### Multivariate Prognostic Validity of Nationwide Subjective and Objective Assessments

In each age group, the overall fits for the subjective [Model 1: 40.87 ≤ $\chi_{\left(4\right)}^{2}$ ≤ 305.20, *p* < 0.001; 0.07 ≤ Nagelkerke's *R*^2^ ≤ 0.11] and objective [Model 2: 40.87 ≤ $\chi_{\left(5\right)}^{2}$ ≤ 388.02, *p* < 0.001; 0.08 ≤ Nagelkerke's *R*^2^ ≤ 0.13] assessment models were significantly better compared to the null model. That is, both the subjective, as well as the objective assessment, significantly predicted the binary outcome (selected vs. non-selected player) in each age group (each *p* < 0.001). Except for the age group U15, where the explained variance by subjective evaluations (i.e., 10%) exceeded those by the objective assessment (i.e., 8%), model 2 comprising the objective diagnostics showed higher values of explained variance compared to model 1 (13, 14, and 13% vs. 10, 11, and 7% for the age groups U12, U13, and U14; for further details, see Supplementary Tables 3a,b).

Moreover, hierarchical regressions revealed that adding the subjective predictors of model 1 to the objective predictors of model 2 [for the age group U12–U14, 42.67 ≤ $\Delta \chi_{\left(4\right)}^{2}$ ≤ 137.29, each *p* < 0.001; 0.04 ≤ Δ Nagelkerke's *R*^2^ ≤ 0.06] or adding the objective predictors of model 2 to the subjective predictors of model 1 [U15; $\Delta \chi_{\left(5\right)}^{2}$ = 20.42, *p* < 0.01; Δ Nagelkerke's *R*^2^ = 0.05] led to a significant increase in the explained variance. Thus, the combination of the subjective and objective assessments (model 3) showed the best statistical explanatory power in every age group [Model 3: 61.29 ≤ $\chi_{\left(9\right)}^{2}$ ≤ 525.31, *p* < 0.001; 0.15 ≤ Nagelkerke's *R*^2^ ≤ 0.20].

The results of the logistic regressions for model 3 separated by age group are displayed in Table 4. Regarding the significance of the predictors, the subjectively evaluated tactical skills as well as the motor test results for sprinting and dribbling significantly contributed to the explanation of players' future success in each age group (each *p* < 0.05). Moreover, kicking skills was a significant predictor for all age groups except for U15 (each *p* < 0.05) and juggling for the younger age groups U12 and U13 (each *p* < 0.01), whereas ball control only showed a significance for the youngest age group U12 (*p* < 0.01). In contrast, the predictors endurance, psychosocial skills, and agility did not show any significant positive contribution to the models in three of the four investigated age groups. In 9 out of 12 cases, the regression coefficients for these three predictors in the four age groups were not significant (each *p* > 0.09). Interestingly, and presumably caused by the noticeable correlations to other explaining variables in the model, in the three remaining cases, psychosocial skills (U12, *p* = 0.02), endurance (U13, *p* < 0.01), and agility (U13, *p* = 0.01) contributed in the opposite, non-expected direction to the explanation of the criterion within the multivariate model.

**Table 4:** Logistic regression results for the prediction of players future success (selected for a YA three seasons later) in dependence of the subjective (printed in italics) and objective assessments (model 3, separated by age group).

**Age group**	**Omnibus tests**			**Predictor**	**Logistic regression coefficients**				**(*e^*b*^*)*^*SD*^* (#)**
	***χ^2^* (*df*)**	***p***	**Nagelkerke *R*^2^**		***b***	**Wald**	***p***	***e^*b*^* (95% CI)**	
U12	525.31 (9)	<0.001	0.17	Constant	12.53	−	−	–	–
				Sprint	−3.49	107.71	<0.01	0.03 (0.02; 0.06)	1.75
				*Tactical skills*	0.87	33.75	<0.01	2.39 (1.78; 3.20)	1.68
				Dribbling	−0.40	23.63	<0.01	0.67 (0.57; 0.79)	1.35
				Juggling	0.05	15.44	<0.01	1.05 (1.02; 1.07)	1.16
				Ball control	−0.13	10.06	<0.01	0.88 (0.81; 0.95)	1.19
				*Kicking skills*	0.39	9.03	<0.01	1.47 (1.14; 1.89)	1.24
				*Psychosocial skills*	−0.25	5.21	0.02	0.78 (0.63; 0.97)	0.85
				Agility (CODS)	0.22	2.29	0.13	1.24 (0.94; 1.64)	–
				*Endurance*	−0.08	0.72	0.40	0.93 (0.78; 1.10)	–
U13	354.65 (9)	<0.001	0.20	Constant	11.97	−	−	–	–
				Sprint	−4.37	111.22	<0.01	0.01 (0.01; 0.03)	2.05
				*Tactical skills*	0.84	20.67	<0.01	2.32 (1.61; 3.34)	1.63
				Juggling	0.05	15.87	<0.01	1.05 (1.03; 1.07)	1.24
				Dribbling	−0.44	14.74	<0.01	0.65 (0.52; 0.81)	1.35
				*Kicking skills*	0.61	13.13	<0.01	1.83 (1.32; 2.54)	1.40
				*Endurance*	−0.39	12.19	<0.01	0.67 (0.54; 0.84)	0.74
				Agility (CODS)	0.52	7.85	0.01	1.68 (1.17; 2.41)	0.82
				Ball control	−0.05	0.76	0.38	0.95 (0.86; 1.06)	–
				*Psychosocial skills*	0.04	0.08	0.78	1.04 (0.79; 1.36)	–
U14	176.87 (9)	<0.001	0.17	Constant	16.13	−	−	–	–
				Sprint	−4.41	69.83	<0.01	0.01 (0.00; 0.03)	2.13
				Dribbling	−0.52	9.98	<0.01	0.59 (0.43; 0.82)	1.40
				*Tactical skills*	0.72	8.06	<0.01	2.05 (1.25; 3.36)	1.52
				*Kicking skills*	0.54	6.17	0.01	1.72 (1.12; 2.64)	1.36
				Ball control	−0.13	3.09	0.08	0.87 (0.75; 1.02)	–
				Juggling	0.02	1.57	0.21	1.02 (0.99; 1.05)	–
				*Psychosocial skills*	−0.20	1.43	0.23	0.82 (0.58; 1.14)	–
				*Endurance*	−0.18	1.35	0.24	0.83 (0.61; 1.13)	–
				Agility (CODS)	0.19	0.64	0.42	1.21 (0.75; 1.96)	–
U15	61.29 (9)	<0.001	0.15	Constant	8.31	−	−	–	–
				*Tactical skills*	1.31	7.75	0.01	3.70 (1.47; 9.29)	2.09
				Dribbling	−0.59	4.21	0.04	0.56 (0.32; 0.97)	1.53
				Sprint	−1.98	3.85	0.05	0.14 (0.02; 1.00)	1.36
				*Psychosocial skills*	−0.52	2.81	0.09	0.60 (0.33; 1.09)	–
				Ball control	−0.23	2.50	0.11	0.79 (0.59; 1.06)	–
				Juggling	0.03	1.99	0.16	1.03 (0.99; 1.08)	–
				*Kicking skills*	0.56	1.98	0.16	1.76 (0.80; 3.85)	–
				*Endurance*	−0.17	0.36	0.55	0.85 (0.49; 1.46)	–
				Agility (CODS)	0.12	0.07	0.80	1.12 (0.46; 2.77)	–

*Predictors were ordered by increasing values with regard to the Wald statistic. (#) In order to facilitate comparisons for effect sizes of individual predictors, the odds ratio coefficients e^b^ were additionally adjusted to the standard deviations of the respective age group (Höner and Votteler, 2016). The resulting (e^b^)^SD^ represent the relative change of the likelihood for being selected for a YA by a one standard deviation increase within the considered predictor. For negatively coded predictors, the adjusted odds ratios were inverted and displayed as (e^b^)^−SD^*.

With regard to the adjusted odds ratios *(e*^*b*^*)*^*SD*^ for significant predictors in the logistic regressions, it can generally be seen that sprint (except for U15) and tactical skills were characterized by the highest scores. For instance, a one standard deviation better result in the sprint would approximately double the chance of being selected for a YA three seasons later in the age groups U12 [*(e*^*b*^*)*^*SD*^ = 1.75], U13 [*(e*^*b*^*)*^*SD*^ = 2.05], and U14 [*(e*^*b*^*)*^*SD*^ = 2.13]. The regression results revealed a similar importance for tactical skills in U15 [*(e*^*b*^*)*^*SD*^ = 2.09], and this performance factor was still a relevant predictor in the younger age groups [1.52 ≤ *(e*^*b*^*)*^*SD*^ ≤ 1.68]. Moreover, dribbling in all age groups [1.35 ≤ *(e*^*b*^*)*^*SD*^ ≤ 1.53] and kicking skills in U13 [*(e*^*b*^*)*^*SD*^ = 1.40] and U14 [*(e*^*b*^*)*^*SD*^ = 1.36] proved their relevance in this regard, whereas all further significant predictors provided limited predictive relevance [*(e*^*b*^*)*^*SD*^ ≤ 1.24].

## Discussion

For a discussion of the study results, it is important first to identify the stage of talent development at which the study took place and second the parameters of the study design used to investigate the talent predictor's prognostic relevance. Regarding the first aspect, Williams et al. (2020) provided a conceptual framework for the talent identification and development process. They identified three key processes for players who are not involved in a structured talent program: detection (i.e., screening for players from outside soccer); participation (i.e., playing soccer but not in a structured development program); and identification (i.e., recognizing soccer players who have the potential to progress in a structured development program). Studies in this field are characterized by a heterogeneous sample (e.g., Hohmann et al., 2018). In contrast, the present study was conducted within the German soccer talent development program, where participants had already been selected as competence center (CC) players leading to a more homogeneous sample. Therefore, according to the conceptual framework, the present results refer to the stages of “development” (i.e., providing the players with a suitable learning environment), “selection” (i.e., on-going process of selecting players within the development program; e.g., for the next highest level or age group) and “deselection” (i.e., removing players from the program) of youth players (Williams et al., 2020).

The present study provides comprehensive insights into empirical evidence for the prognostic validity of nationwide assessments and thus of subjectively and objectively assessed performance factors in youth elite soccer. First, within a *univariate perspective*, the results confirmed the prognostic validity for all considered predictors addressing the motor, perceptual–cognitive, and personality-related domain. Thereby, the variables with the highest predictive value (i.e., tactical skills, kicking skills, and sprint) represent both assessment methods (subjective and objective) and depict different domains. However, effect sizes were limited, and thus, the sensitivity was not high enough to justify case-by-case selection decisions on the stand-alone basis of these assessments (Carling and Collins, 2014; Höner and Votteler, 2016). Second, within a *multivariate perspective*, the results provided empirical evidence for the subjective and objective assessments' prognostic validity. Overall, the objective assessment showed higher explanatory power when comparing both assessments. Most important, the combined model, integrating both assessments, revealed explained variances of 15% ≤ Nagelkerke's *R*^2^ ≤ 20% over the four investigated age groups and proved superior to the models comprising either the subjective or objective assessment. Consequently, this addresses current gaps within the literature by linking subjective coach evaluations with objective measures (Williams et al., 2020). Within these multidisciplinary models, sprint, tactical skills, and dribbling were found to be the most predictive variables, although the order of the most significant predictors varied in parts among the age groups.

### Prognostic Validity of the Performance Factors

The present study analyzed large-scale nationwide assessments based on a powerful sample size and assessed data from U12, U13, U14, and U15 players belonging to the top 4% of their age group in Germany. The prospective criterion was the achievement of youth academy status (i.e., top 1% of all players) three seasons later, with a success rate of 9%. For a more detailed interpretation, one must take into consideration the variety of *study design features* in prospective studies that influence the expectable amount of significant effects and their sizes (Hohmann et al., 2018; Bergkamp et al., 2019). For instance, players' age and soccer development stage (e.g., foundation, talent, or elite stage), the level and type of the criterion variable, and the applied assessments must be considered as moderator variables regarding the prognostic relevance of talent predictors (Murr et al., 2018b). Moreover, due to the sports-specific performance requirements, important information with respect to prognostic validity such as effect sizes are only comparable within a specific sport.

Therefore, the following discussion about the effect sizes presented in this study is focused on recent systematic reviews exploring prospective studies in youth soccer (such as Murr et al., 2018a,b; Sarmento et al., 2018; Williams et al., 2020) because reviews provide “overarching approaches” and are not dependent on each single study design feature. Moreover, individual studies with similar design features were considered for comparing effect sizes to the present study (and if these studies provided effect sizes other than Cohen's *d, d* was reanalyzed from the presented descriptive statistics). Studies from three nationwide talent development programs were considered. First, studies investigating male players from the German program at these age groups (Höner and Feichtinger, 2016; Höner and Votteler, 2016) seem to be most comparable to the present results (e.g., because of similar success rate). Second, two prospective studies conducted within the Swiss talent development program (Sieghartsleitner et al., 2019a,b) used similar diagnostic tools to assess motor performance factors (i.e., the same tests for agility, dribbling, juggling, and a modified version for ball control) and were characterized by further similar designs parameters. For example, the Swiss program selects the top 6% of the registered U12 players in their early selections for talent bases and about 1% for their elite youth development program in U15 (Romann and Fuchslocher, 2013). Grounding on this, the success rate in the prospective studies for a U13/U14 sample was 12%, and for a U14 sample, 17% (Sieghartsleitner et al., 2019a). However, these studies were characterized by less statistical power due to a smaller sample size (*N* ≤ 133), leading to a lower sensitivity to detect small effect sizes. Third, Saward et al. (2020) published a longitudinal study with data from male soccer players between 8 and 19 years from the English talent development system program. The data for the age groups U12–U15 was also considered suitable for a comparison with the present study results. In each age group, Saward et al. (2020) investigated about 1,000 players from 16 professional academies in England. As the prospective success rate of these players was between 17 and 30% for the age groups U12–U15, this might indicate a more elite sample compared to the present study.

#### Physiological Abilities (Motor Domain)

Regarding potential physiological talent predictors, this study investigated linear and change in direction speed abilities (i.e., sprint, agility) and endurance. According to the systematic review conducted by Murr et al. (2018b), mid-term prospective studies in early adolescence provided mixed results regarding the prognostic validity of *agility* [change of direction speed (CODS)] tests. For age groups U12–U15, there is a noticeable variation in the effect sizes across age groups ranging from non-significant effects (Deprez et al., 2015) to significant and large effect sizes of more than one standard deviation (Figueiredo et al., 2009). Presumably, due to its large sample size, the present study provided more robust results regarding the effect sizes. Here, agility varied only to a small extent around the median *d* = 0.30. This effect size is in line with the general conclusion in the review conducted by Williams et al. (2020, p. 3) that future successful players are “slightly more agile.” Similarly, a prospective study conducted by Höner and Votteler (2016) found not only significant but also small effect sizes for agility with CC players from earlier birth cohorts (i.e., 1993–1997). Saward et al. (2020) revealed significantly better agility performance in future professional players; however, the effect sizes were trivial to small in adolescence (*d* < 0.20 by the age 12.0) and got larger with further development (*d* = 0.50 by the age 18.0). In contrast, Sieghartsleitner et al. (2019a) descriptively presented agility test results of Swiss U14 players that did not indicate any differences (*d* < 0.10) between players who achieved a professional level 5 years later or those who did not.

In relation to *sprint* performance, Saward et al. (2020) found that future professional players in England were faster in a 20-m sprint than non-professional players but only reported a small effect size (*d* = 0.20 over all age groups, U9–U20). In the present study, the 20-m sprint was one of the most relevant tests and revealed the highest effect size of *d* = 0.73 for the age group U14, whereas Sieghartsleitner et al. (2019a) did not find prognostic relevance for a 40-m sprint test for the same age group. This inconsistency may be caused by the diverse sprinting distances in the study, although in the review by Murr et al. (2018b), sprinting tests with a distance of >20 m were more relevant than the tests with shorter distances (≤20 m). Going beyond, further inconsistencies related to study design, such as performance level of players and length of prognostic periods, may also impact the generalizability of the study results. For example, Sieghartsleitner et al. (2019a) as well as Saward et al. (2020) utilized longer prognostic periods (≥5 years). This, together with partly different performance levels of players at the time of predictors' assessment (i.e., a higher level of selection, Saward et al., 2020), might have led to lower effect sizes detected in these studies compared to the present study. Although recent reviews (Murr et al., 2018b; Williams et al., 2020) and the present study provide insight into the predictive power of sprint performance, future research is needed to clarify whether sprint results may be less relevant regarding predictive validity in older aged or higher selected groups. At least, there seems to be a trend in this direction since, in the present study, the effect size for U15 (*d* = 0.40; *p* < 0.001) was remarkably smaller than for the younger age groups (although still significant). Moreover, Murr et al. (2018b) found less significant empirical evidence for sprinting tests in the elite stage (U16–U19) compared to the talent stage (U12–U15).

With regard to the assessment of *endurance*, it should be noted that this performance factor was evaluated subjectively by coaches. Dugdale et al. (2020) indicate that coaches reach their limits in subjective evaluations when players show similar performances regarding endurance. Nevertheless, in the present study, the mean univariate effect size of *d* = 0.43 is within the range of the significant effect sizes presented in the review by Murr et al. (2018b) for objective endurance diagnostics (0.28 ≤ *d* ≤ 1.56). However, that review also revealed that nearly one-third of the effect sizes identified for endurance in the talent stage phase (U12–U15) were non-significant, and in a more recent study, Sieghartsleitner et al. (2019a) found the (objective) Yo-Yo intermittent recovery test in U14 age group not to be of predictive relevance for future success. Moreover, besides the different approaches of assessing endurance (i.e., subjective vs. objective), different statistical approaches may lead to a further inconsistency regarding the prognostic validity. For example, when utilizing a multivariate approach, Sieghartsleitner et al. (2019a) found a negative relationship with professional player status (i.e., Swiss U19 junior national players) for their comprehensive general motor performance variable (i.e., Yo-Yo intermittent recovery test, counter movement jump, 40 m linear sprint, and agility test). In a similar way, the multivariate analysis in the present study revealed in each of the considered age groups negative effects (i.e., *e*^*b*^ < 1; in one case significant: *p* < 0.01 for U13) for the subjectively rated endurance item when considered in combination with the other performance factors. Although multicollinearity between the predictors of the logistic regression models was acceptable in terms of *VIF*, the different results for endurance in the univariate and multivariate perspective are likely due to noticeable correlations with other (mainly subjective) predictors. Therefore, more research regarding the prognostic validity of this factor, predominantly in multidimensional approaches, is needed.

#### Technical Skills (Motor Domain)

In addition to the reviews for sport (Sarmento et al., 2018; Koopmann et al., 2020) or soccer skills (Murr et al., 2018a; Williams et al., 2020), the present study underlines the importance of technical skills. The median effect size for *dribbling* (*d* = 0.47) in this study was at the lower end of the range of effect sizes that Murr et al. (2018a) identified in 10 prospective soccer studies (0.47 ≤ *d* ≤ 1.24). Interestingly, *ball control* (*d* = 0.34) was below the range (0.57 ≤ *d* ≤ 1.28) detected by Murr et al. (2018a). Moreover, *juggling*, a skill not investigated as much as other technical skills, proved to be a significant predictor with a median effect size of *d* = 0.42 and was also found to be a relevant predictor in the younger age groups (i.e., *d* = 0.52 in U12 compared to *d* = 0.35 in U14). In both studies within the Swiss program, even higher effect sizes were detected regarding juggling compared to the present study. While Sieghartsleitner et al. (2019a) found a medium effect size (*d* = 0.62) for the U14 age group, in a second study with a comparable sample of U14 soccer players, a large effect size (*d* = 1.07) was found (Sieghartsleitner et al., 2019b). However, these inconsistencies regarding the detected effects in these studies could have been affected by the rather low sample size of players in the professional groups (17 ≤ *n* ≤ 20).

Of particular interest, especially when considering a direct comparison of objective and subjective assessments, are the results for *kicking skills*, as these results can be compared to the prognostic validity of a shooting test investigated by Höner and Votteler (2016). Although practitioners consider shooting skills to be very important for soccer performance, only small effect sizes were found in that study. However, a challenge for researchers is the ability to create a reliable and valid shooting skill test, and many tests have poor psychometric properties (Ali, 2011; Höner et al., 2015). Therefore, within the German talent development program, the shooting test was substituted by the subjective rating presented in this study. Indicated by an effect size of *d* = 0.71, the newly established subjective evaluation of kicking skills proved to have superior prognostic validity compared to the former shooting test. One reason for this may be that the subjective assessment of kicking skills was based on a broader concept compared to the objective shooting skill tests where only the precision and speed of the shots were registered. For example, coaches evaluate the ability of the CC players to use different kicking techniques, to pass or shoot at the goal with a high degree of variability, at a speed appropriate to the game situation or under pressure from the opponent (see Supplementary Table 1a).

#### Individual Tactical Skills (Perceptual–Cognitive Domain)

*Tactical skills* such as behavior in one vs. one offensive/defensive situations, anticipation, decision-making, or game intelligence have been found to be crucial for achieving top-level performance in football (e.g., Roca et al., 2012). In accordance to the current results, Murr et al. (2018a) identified in their systematic review medium to large effect sizes for the prognostic relevance of these skills. However, the effects sizes are based on only four prospective studies, indicating a lack of such studies and limiting the empirical evidence for perceptual–cognitive skills compared to other domains. Moreover, the review identified only one study in soccer to assess these skills based on an objective assessment. O'Connor et al. (2016) used video-based tests including different soccer-specific tasks of tactical skills (e.g., decision-making), which prompt a written response. Such video-based diagnostic instruments are established in the (cross-sectional) expertise research; however, they are considered to have limitations regarding the representativeness of the stimuli presentation as well as the limited motor response of the participants (e.g., Travassos et al., 2013). Besides critical arguments regarding the representativeness of video-based diagnostics, test economic reasons might cause the lack of prospective studies examining the predictive value of perceptual–cognitive factors. For example, three studies (i.e., Kannekens et al., 2011; Huijgen et al., 2014; Forsman et al., 2016) providing empirical evidence in the systematic review used the tactical skill inventory in which players rate their soccer performance in comparison to the top players in the same age category (TACSIS; Elferink-Gemser et al., 2004). However, besides general criticisms concerning the lack of soccer-specific performance response (Nortje et al., 2014), self-reported tactical skills might be biased by socially desired answers or unrealistic self-concept.

To avoid such biased assessments, Williams et al. (2020) suggested that researchers should consider measuring individual performance factors, *via* coach ratings, in matches or small-sided games. However, the authors acknowledged the challenge of developing rating tools with satisfactory psychometric properties. The present study revealed excellent reliabilities for the subjective evaluation tool. Going beyond, tactical skills were the strongest predictor in the univariate (0.61 ≤ *d* ≤ 0.85) and one of the strongest in the multivariate analyses. Therefore, the tactical skills evaluation tool (Table 1) proved to be an appropriate assessment method within a nationwide talent development program. While a positive outcome, future research is needed to explore the correlations between the coaches' ratings to other perceptual–cognitive diagnostics such as in-game performance assessments (e.g., small-sided games; Bergkamp et al., 2020), video-based decision-making skills tests with a soccer-specific response (Murr et al., 2021), or assessments of general cognitive functions (e.g., Beavan et al., 2020).

#### Psychosocial Skills (Personality-Related Domain)

Comprehensive models for talent development (e.g., DMGT; Gagné, 2010) provide a theoretical basis for examining the predictive value of psychosocial dispositions and skills from the personality-related domain, such as motivation or volitional self-regulation (e.g., Mills et al., 2012) that are mainly investigated with subjective self-report questionnaires. Several systematic reviews (e.g., Gledhill et al., 2017; Murr et al., 2018a; Ivarsson et al., 2020; Williams et al., 2020) highlight the importance of psychological predictors for (successful) talent development in soccer. Nevertheless, similar to the other investigated domains in this study, the effect sizes in these reviews do not support the use of sport psychological diagnostics as a selection tool in talent development programs.

This is also in line with the present results regarding the psychosocial skills, although the median effect size (*d* = 0.43) represents a recognizable prognostic relevance that was, to some extent, larger than the effects found in a comparable study within the German talent development program (Höner and Feichtinger, 2016). In that 4-year prospective study, U12 players (*N* = 2,677) completed 17 psychological scales of established sport psychological self-report questionnaires addressing motivational, volitional, self-referential cognitions and emotional characteristics. Whereas the majority of the psychological scales (10 out of 17) proved to be significant in predicting success (i.e., achieving the youth academy level at U16), the effect sizes were smaller (0.19 ≤ *d* ≤ 0.30) than in the present study. This indicates that coaches' subjective assessment of psychological characteristics may add explanatory power to self-report questionnaires (Musculus and Lobinger, 2018) because coaches can base their judgements on observations of a player's behavior in several “representative” situations and in reference to the other talented players. However, further research is needed to bridge the gap between the (scientifically sound) sport psychological self-report questionnaires and the (often unevaluated) scouting sheets occasionally used in youth soccer. Thereby, recommendations on how to improve the subjective assessments of personality-related factors and how to educate the youth soccer coaches in this regard should be considered (Musculus and Lobinger, 2018).

### Limitations and Perspectives

Although the present study is characterized by several strong design features (i.e., homogeneous sample with talented players, high test power, midterm prognostic period, and multidimensional subjective and objective assessment), several limitations need to be considered. First, in accordance with the vast majority of studies in this research area, the current study focused on assessing performance at a *single time point*. However, based on the multidimensional and dynamic conceptualization of talent (Buekers et al., 2015) and, in some parts, the inconsistent results in longitudinal studies (e.g., Leyhr et al., 2018; Saward et al., 2020), further empirical studies are needed to examine the progress of players' performance factors longitudinally. However, it should be noted that the present study focused on the simultaneous examination of the validity of subjective and objective assessments in an attempt to address the gap in this field of research. Moreover, from a methodological perspective, there are some non-trivial difficulties when combining subjective and objective assessments in one longitudinal analysis. For example, in this study, the coaches were asked to evaluate the players' performance factors in regards to the corresponding reference groups (Ivarsson et al., 2020). Thus, in contrast to the motor tests, no absolute values were available from the implemented subjective assessment and an investigation of players' developmental process would have been restricted to the exploration of development only relative to other players.

Second, the *operationalization of the criterion variable* is a methodological issue critically debated in soccer talent identification research (Bergkamp et al., 2019). The prognostic period in this study ranged from early to middle adolescence, whereas the criterion was operationalized as performance level in age groups U15, U16, U17, and U18 respectively. As juvenile success as an appropriate predictor for success in adulthood might be questioned in sport (Güllich and Emrich, 2014), this prediction period may be regarded as a limitation. However, being selected for the youth academies (that is, the present study's criterion variable) seems to be strongly associated with success in adulthood in German soccer, as nearly 90% of German adult professional players played at least one season for a youth academy (Güllich, 2014). Furthermore, the criterion for the prediction was operationalized as performance level. Bergkamp et al. (2019, p. 1319), highlighted “an explicit measure of soccer performance is rarely used as a criterion,” and this may imply some problems. For instance, binary-coded performance levels, as utilized in this study, provide only limited information on the individual differences between players' soccer performance. However, such information is helpful as decisions on selecting or deselecting players frequently occur in sport talent pathways (Ford et al., 2020), and especially in the present study over a prolonged prognostic period of three seasons, different coaches and institutions (competence centers, regional associations, youth academies, youth national teams) were involved in these decisions concerning the players. Moreover, valid and test economic assessments for measuring individual soccer performance in large-scale studies are challenging. Consequently, an operationalization of soccer performance by players' performance level seems appropriate from a practical perspective (Bergkamp et al., 2019).

Third, the players' development and their performance factors are not only dynamic but also interactive with additional factors. Thereby, not only personal but also *environmental factors*, such as family support or training history, play a decisive role. However, one of the few studies investigating the combination of motor performance diagnostics and coaches' subjective evaluations with additional information about family support and training history led to the conclusion that including these environmental factors in existing data on motor performance tests and coach assessments did not provide significant additional explanatory power (Sieghartsleitner et al., 2019a). Therefore, the integration of environmental factors in prognostic models may be a demanding task for future studies (e.g., when considering the operationalization of the variables and the underlying assumptions about the association between constructs such as family support and successful player development). That is, probably an optimal but not maximal family support may be most supportive for a player, and going beyond, this optimum might be individualized and thus different for each player.

Moreover, further *potential moderating or confounding variables* were not in the scope of this study. For example, Sarmento et al. (2018) highlighted a complex relationship between youth players' performance factors according to relative age, maturity status, or specific playing positions. In particular, maturational status might have enabled interesting insights for the present study, as indicated by a recent study from Hill et al. (2020b), who observed positive associations between coaches' subjective match performance ratings and maturation in U14 and U15 academy soccer players. However, data on maturity status was not assessed in this large-scale study, and assessments such as the Mirwald method (Mirwald et al., 2002) or the Khamis–Roche method (Khamis and Roche, 1994) still need further investigation concerning their reliability and validity (Myburgh et al., 2019; Leyhr et al., 2020a). Regarding information about relative age, interactions between future success and relative age for each investigated predictor were found non-significant, and therefore, this was not considered as confounder for the present examination of prognostic validity. Nevertheless, future research should investigate relative age-related biases regarding objective (e.g., Votteler and Höner, 2017; Hill et al., 2020a) and subjective assessments (e.g., Furley and Memmert, 2016). In addition, talent predictors' prognostic relevance with respect to specific playing positions seems to be a promising perspective. As specific playing positions within players' talent development in the investigated age groups (U12–U15) may frequently change (e.g., especially considering their future playing position in professional soccer), data about specific playing positions were not assessed and considered in the present study. However, future research should address this when investigating predictors assessed in later stages of talent development and their predictive value for reaching a professional level in soccer.

A further limitation is that this study only investigated male players. With a few exceptions (Datson et al., 2020; Leyhr et al., 2020b), this is typical of talent research in sport, with an overrepresentation of male studies in general (Johnston et al., 2018; Koopmann et al., 2020) and in soccer (Murr et al., 2018b; Sarmento et al., 2018). However, *gender* must be considered as a potential moderator variable regarding the prognostic relevance of talent predictors, and thus, future studies should extend their focus to female athletes (Williams et al., 2020), in particular due to the increasing popularity of female soccer (Manson et al., 2014).

### Conclusion and Implications for Practice

In conclusion, this study provides empirical evidence for the prognostic validity of performance factors from different domains and thus reinforces the call for multidimensional assessments and the use of objective as well as subjective assessments in talent development programs (Sieghartsleitner et al., 2019a; Williams et al., 2020). All nine investigated predictors proved to be significant, but integrating the univariate and the multivariate perspective, tactical skills, and sprint, and thereafter kicking skills and dribbling, were the most important predictors in this study.

Interestingly, the univariate predictive power of the four subjectively rated performance factors were detected to be slightly higher than those of the five objectively assessed motor performance factors. However, multivariate results indicated a higher explanatory power of the objective assessment compared to the subjective assessment. As a consequence, predictors such as kicking skills and psychosocial skills proved more relevant in the univariate than in the multivariate perspective. This may be due to the breadth and dominance of the tactical skills in the subjective assessments and also suggests that coaches' subjective ratings differentiate less between theoretical distinct constructs than objective assessments. At least, it is remarkable that the subjective assessments in this study addressed a broader spectrum of performance factors compared to the objective assessments but lost more explanatory power in the multivariate analyses. This may be interpreted as a weakness of the subjective assessment, and coaches should be educated regarding their analytic skills of performance factors. On the other hand, the subjective assessment also proved strengths in assessing more complex skills like tactical, kicking, or psychosocial skills that are difficult to assess *via* objective (and test economic) measures in nationwide programs. Thus, a challenge for future research is to explore strengths and weaknesses of both assessment approaches and the optimal method to integrate them.

In regard to practical implications, it is important to note that both subjective and objective assessments in the German talent development program mainly support the monitoring of players' development and are therefore not used as a “tool” for selection or deselection. Accordingly, CC coaches are advised to base their selection decisions on their personal experiential knowledge. Nevertheless, the assessments may provide an additional piece of information for making these decisions. More importantly, to know which factors are relevant for the development of successful players, the presented empirical evidence for the prognostic relevance of the investigated factors is crucial.

Moreover, from an organizational perspective, the study results may also inspire youth academies or soccer associations coach education programs to develop about the coaches “eye” for relevant talent predictors. First, by describing performance factors such as kicking skills, endurance ability, tactical skills, and psychosocial skills in a detailed manual provides a “common” understanding of these factors, and coaches can evaluate their players as uniformly as possible in the nationwide program. Second, by implementing the subjective assessments, CC coaches were consciously encouraged to deal with the described talent predictors and to discuss them with their peers or with the associations staff members responsible for CC coach education programs. Furthermore, CC coaches are able to evaluate and reflect on their diagnostic ratings and discuss this with peers or association staff members. Thereby, their documented views may be strengthened if the CC coaches' evaluations are confirmed by players' future success; however, if there is a difference, specific education may be offered. For this purpose, the manual may also provide the foundation and the described key points, and their explanations can be used to create instructional videos that allow CC coaches to convey a uniform idea of the individual talent characteristics.

## Data Availability Statement

The raw data supporting the conclusions of this article will be made available by the authors, without undue reservation.

## Ethics Statement

The studies involving human participants were reviewed and approved by Ethics Committee of the Faculty of Social Sciences and Economics, University of Tübingen. Written informed consent to participate in this study was provided by the participants' legal guardian/next of kin.

## Author Contributions

OH, DL, and DM: conceptualization and methodology. OH: data curation, supervision, funding acquisition, project administration, validation, visualization, and writing original draft. DL: formal analysis. OH, DL, DM, and RS: investigation. OH, DL, DM, RS, and PL: writing review and editing. All authors contributed to the article and approved the submitted version.

## Conflict of Interest

The authors declare that the research was conducted in the absence of any commercial or financial relationships that could be construed as a potential conflict of interest.

## Footnotes

^1^There are inconsistent definitions of agility in sport science literature (Sheppard et al., 2014). In the present study, the term “agility” is used for tests that assess a speed-related motor ability enabling preplanned changes of movement direction that does not include cognitive aspects such as anticipation or decision-making. Thus, this study investigated the physical dimension (or movement component) of agility. This perspective categorizes agility as a physical performance factor in accordance with the majority of prospective studies referred to in the present article (e.g., Hohmann et al., 2018; Sieghartsleitner et al., 2019b; Saward et al., 2020) as well as with the recent reviews on talent identification research in soccer (e.g., Bergkamp et al., 2019; Williams et al., 2020). However, following the work by Sheppard and Young (2006), agility is often used in a broader sense whereby agility is considered an open skill based on two factors, “change of direction speed” (CODS) and perceptual–cognitive facets such as anticipation and decision-making. To address the inconsistency in the literature, we use both terms in the figures and tables of the present study [i.e., “Agility (CODS)”] to indicate that we refer to the physical dimension of agility but not to the more complex reactive agility. In a recent position paper, Young et al. (2021) encourage researchers to study (reactive) agility in more ecologically valid settings that include a sport-specific stimulus (e.g., video simulations, 1 vs. 1 situations, small-sided games). This call is also true for the other objective motor diagnostics investigated in the present study because they do not represent the contextual constraints of soccer games (for a discussion of the representativeness and fidelity of predictors in talent identification research in soccer, see also Bergkamp et al., 2019).

^2^In addition, to have a pragmatic measure for the chance of players' future success, groups of low [percentile rank (*PR*) ≤ 33.33], medium (33.33 < *PR* ≤ 66.67), and high (*PR* > 66.67) performers were built for each considered predictor. Based on these categorizations, odds ratios being selected for a YA for players with high vs. medium and high vs. low performance outcomes were calculated (results for these analyses are presented in the Supplementary Table 2).

## References

[B1] Ackerman P. (2014). Nonsense, common sense, and science of expert performance: talent and individual differences. Intelligence 45, 6–17. 10.1016/j.intell.2013.04.009

[B2] Akinwande O. Dikko H. Agboola S. (2015). Variance inflation factor: as a condition for the inclusion of suppressor variable(s) in regression analysis. Open J. Stat. 05:14. 10.4236/ojs.2015.57075

[B3] Ali A. (2011). Measuring soccer skill performance: a review. Scand. J. Med. Sci. Sports. 21, 170–183. 10.1111/j.1600-0838.2010.01256.x 21210855

[B4] Baláková V. Boschek P. Skalíková L. (2015). Selected cognitive abilities in elite youth soccer players. J. Hum. Kinetics 49, 267–276. 10.1515/hukin-2015-0129 26839627 PMC4723177

[B5] Beavan A. Spielmann J. Mayer J. Skorski S. Meyer T. Fransen J. (2020). The rise and fall of executive functions in high-level football players. Psychol. Sport Exerc. 49:101677. 10.1016/j.psychsport.2020.101677 32711397

[B6] Bergkamp T. den Hartigh R. Frencken W. Niessen A. Meijer R. (2020). The validity of small-sided games in predicting 11-vs-11 soccer game performance. PLoS ONE 15:e0239448. 10.1371/journal.pone.0239448 32956368 PMC7505454

[B7] Bergkamp T. Niessen A. den Hartigh R. Frencken W. Meijer R. (2018). Comment on: “talent identification in sport: a systematic review”. Sports Med. 48, 1517–1519. 10.1007/s40279-018-0868-6 29429139

[B8] Bergkamp T. Niessen A. den Hartigh R. Frencken W. Meijer R. (2019). Methodological issues in soccer talent identification research. Sports Med. 49(9):1317–1335. 10.1007/s40279-019-01113-w 31161402 PMC6684562

[B9] Breitbach S. Tug S. Simon P. (2014). Conventional and genetic talent identification in sports: will recent developments trace talent? Sports Med. 44, 1489–1503. 10.1007/s40279-014-0221-7 25015477

[B10] Buekers M. Borry P. Rowe P. (2015). Talent in sports. Some reflections about the search for future champions. Mov. Sport Sci. Sci. Motricité 88, 3–12. 10.1051/sm/2014002

[B11] Carling C. Collins D. (2014). Comment on “Football-specific fitness testing: adding value or confirming the evidence?”. J. Sports Sci. 32, 1206–1208. 10.1080/02640414.2014.898858 24878103

[B12] Christensen M. (2009). “An eye for talent”: talent identification and the “practical sense” of top-level soccer coaches. Sociol. Sport J. 26, 365–382. 10.1123/ssj.26.3.365

[B13] Cohen J. (1992). A power primer. Psychol. Bull. 112:155. 10.1037/0033-2909.112.1.155 19565683

[B14] Datson N. Weston M. Drust B. Gregson W. Lolli L. (2020). High-intensity endurance capacity assessment as a tool for talent identification in elite youth female soccer. J. Sports Sci. 38, 1313–1319. 10.1080/02640414.2019.1656323 31451097

[B15] Dawes R. Faust D. Meehl P. (1989). Clinical versus actuarial judgment. Science 243, 1668–1674. 10.1126/science.2648573 2648573

[B16] Den Hartigh R. Niessen A. Frencken W. Meijer R. (2018). Selection procedures in sports: improving predictions of athletes' future performance. Euro. J. Sport Sci. 18, 1191–1198. 10.1080/17461391.2018.1480662 29856681

[B17] Deprez D. Buchheit M. Fransen J. Pion J. Lenoir M. Philippaerts R.. (2015). A longitudinal study investigating the stability of anthropometry and soccer-specific endurance in pubertal high-level youth soccer players. J. Sports Sci. Med. 14, 418–426. PMID: 25983593 25983593 PMC4424473

[B18] Deutscher Fußball Bund (2009). Talentförderprogramm. Leitfaden für Die Ausbildung [Talent Development Programme. Training Guidline]. Münster: Philippka.

[B19] Dugdale J. Sanders D. Myers T. Williams A. Hunter A. (2020). A case study comparison of objective and subjective evaluation methods of physical qualities in youth soccer players. J. Sports Sci. 38: 1–9. 10.1080/02640414.2020.1766177 32536323

[B20] Elferink-Gemser M. Huijgen B. Coelho e Silva M. Lemmink K. Visscher C. (2012). The changing characteristics of talented soccer players – a decade of work in Groningen. J. Sports Sci. 30, 1581–1591. 10.1080/02640414.2012.725854 23020141

[B21] Elferink-Gemser M. Visscher C. Richart H. Lemmink K. (2004). Development of the tactical skills inventory for sports. Percept. Mot. Skills 99, 883–895. 10.2466/pms.99.3.883-895 15648483

[B22] Figueiredo A. Gonçalves C. Coelho-e-Silva M. Malina R. (2009). Characteristics of youth soccer players who drop out, persist or move up. J. Sports Sci. 27, 883–891. 10.1080/02640410902946469 19629837

[B23] Ford P. Bordonau J. Bonanno D. Tavares J. Groenendijk C. Fink C.. (2020). A survey of talent identification and development processes in the youth academies of professional soccer clubs from around the world. J. Sports Sci. 38, 1269–1278. 10.1080/02640414.2020.1752440 32378447

[B24] Forsman H. Blomqvist M. Davids K. Liukkonen J. Konttinen N. (2016). Identifying technical, physiological, tactical and psychological characteristics that contribute to career progression in soccer. Int. J. Sports Sci. Coach. 11, 505–513. 10.1177/1747954116655051

[B25] Furley P. Memmert D. (2016). Coaches' implicit associations between size and giftedness: implications for the relative age effect. J. Sports Sci. 34, 459–466. 10.1080/02640414.2015.1061198 26096053

[B26] Gagné F. (2010). Motivation within the DMGT 2.0 framework. High Ability Stud. 21, 81–99. 10.1080/13598139.2010.525341

[B27] Gil S. Irazusta J. Bidaurrazaga-Letona I. Aduna B. Lekue J. Santos-Concejero J.. (2014). Talent identification and selection process of outfield players and goalkeepers in a professional soccer club. J. Sports Sci. 32, 1931–1939. 10.1080/02640414.2014.964290 25429718

[B28] Gledhill A. Harwood C. Forsdyke D. (2017). Psychosocial factors associated with talent development in football: a systematic review. Psychol. Sport Exerc. 31, 93–112. 10.1016/j.psychsport.2017.04.002

[B29] Güllich A. (2014). Selection, de-selection and progression in German football talent promotion. Euro. J. Sport Sci. 14, 530–537. 10.1080/17461391.2013.858371 24245783

[B30] Güllich A. Emrich E. (2014). Considering long-term sustainability in the development of world class success. Eur. J. Sport Sci. 14, 383–397. 10.1080/17461391.2012.706320 24444233

[B31] Hill M. Scott S. Malina R. McGee D. Cumming S. (2020a). Relative age and maturation selection biases in academy football. J. Sports Sci. 38, 1359–1367. 10.1080/02640414.2019.1649524 31366286

[B32] Hill M. Scott S. McGee D. Cumming S. (2020b). Are relative age and biological ages associated with coaches' evaluations of match performance in male academy soccer players? Int J Sports Sci Coach. 16(2):227–235. 10.1177/1747954120966886

[B33] Hoare D. Warr C. (2000). Talent identification and women's soccer: an Australian experience. J. Sports Sci. 18, 751–758. 10.1080/02640410050120122 11043900

[B34] Hohmann A. Siener M. He R. (2018). Prognostic validity of talent orientation in soccer. German J Exerc Sport Res. 48, 478–488. 10.1007/s12662-018-0549-5

[B35] Höner O. Feichtinger P. (2016). Psychological talent predictors in early adolescence and their empirical relationship with current and future performance in soccer. Psychol. Sport Exerc. 25, 17–26. 10.1016/j.psychsport.2016.03.004

[B36] Höner O. Leyhr D. Kelava A. (2017). The influence of speed abilities and technical skills in early adolescence on adult success in soccer: a long-term prospective analysis using ANOVA and SEM approaches. PLoS ONE 12:e0182211. 10.1371/journal.pone.0182211 28806410 PMC5555567

[B37] Höner O. Votteler A. (2016). Prognostic relevance of motor talent predictors in early adolescence: a group- and individual-based evaluation considering different levels of achievement in youth football. J. Sports Sci. 34, 2269–2278. 10.1080/02640414.2016.1177658 27148644

[B38] Höner O. Votteler A. Schmid M. Schultz F. Roth K. (2015). Psychometric properties of the motor diagnostics in the German football talent identification and development programme. J. Sports Sci. 33, 145–159. 10.1080/02640414.2014.928416 24949838

[B39] Huijgen B. Elferink-Gemser M. Lemmink K. Visscher C. (2014). Multidimensional performance characteristics in selected and deselected talented soccer players. Euro. J. Sport Sci. 14, 2–10. 10.1080/17461391.2012.725102 24533489

[B40] Ivarsson A. Kilhage-Persson A. Martindale R. Priestley D. Huijgen B. Ardern C.. (2020). Psychological factors and future performance of football players: a systematic review with meta-analysis. J Sci Med Sport 23, 415–420. 10.1016/j.jsams.2019.10.021 31753742

[B41] Johnston K. Wattie N. Schorer J. Baker J. (2018). Talent identification in sport: a systematic review. Sports Med. 48, 97–109. 10.1007/s40279-017-0803-2 29082463

[B42] Kannekens R. Elferink-Gemser M. Visscher C. (2011). Positioning and deciding: key factors for talent development in soccer. Scand. J. Med. Sci. Sports 21, 846–852. 10.1111/j.1600-0838.2010.01104.x 22126715

[B43] Khamis H. J. Roche A. F. (1994). Predicting adult stature without using skeletal age: the Khamis-Roche method. Pediatrics 94(4 Pt 1), 504–507. 7936860

[B44] Koopmann T. Faber I. Baker J. Schorer J. (2020). Assessing technical skills in talented youth athletes: a systematic review. Sports Med. 50, 1593–1611. 10.1007/s40279-020-01299-4 32495253 PMC7441090

[B45] Koz D. Fraser-Thomas J. Baker J. (2011). Accuracy of professional sports drafts in predicting career potential. Scand. J. Med. Sci. Sports. 22, e64–69. 10.1111/j.1600-0838.2011.01408.x 22092367

[B46] Larkin P. Marchant D. Syder A. Farrow D. (2020). An eye for talent: the recruiters' role in the Australian Football talent pathway. PLoS ONE 15:e0241307. 10.1371/journal.pone.0241307 33137113 PMC7605670

[B47] Larkin P. O'Connor D. (2017). Talent identification and recruitment in youth soccer: recruiter's perceptions of the key attributes for player recruitment. PLoS ONE 12:e0175716. 10.1371/journal.pone.0175716 28419175 PMC5395184

[B48] Leyhr D. Kelava A. Raabe J. Höner O. (2018). Longitudinal motor performance development in early adolescence and its relationship to adult success: an 8-year prospective study of highly talented soccer players. PLoS ONE 13:e0196324. 10.1371/journal.pone.0196324 29723200 PMC5933705

[B49] Leyhr D. Murr D. Basten L. Eichler K. Hauser T. Lüdin D.. (2020a). Biological maturity status in elite youth soccer players: a comparison of pragmatic diagnostics with magnetic resonance imaging. Front. Sports Active Living 2:587861. 10.3389/fspor.2020.587861 33345157 PMC7739788

[B50] Leyhr D. Raabe J. Schultz F. Kelava A. Höner O. (2020b). The adolescent motor performance development of elite female soccer players: a study of prognostic relevance for future success in adulthood using multilevel modelling. J. Sports Sci. 38, 1342–1351. 10.1080/02640414.2019.1686940 31680634

[B51] Lund S. Söderström T. (2017). To see or not to see: talent identification in the Swedish Football Association. Sociol. Sport J. 34, 248–258. 10.1123/ssj.2016-014432433076

[B52] MacMahon C. Bailey A. Croser M. Weissensteiner J. (2018). Exploring the skill of recruiting in the Australian Football League. Int. J. Sports Sci. Coach. 14, 72–81. 10.1177/1747954118809775

[B53] Manson S. Brughelli M. Harris N. (2014). Physiological characteristics of International Female Soccer Players. J. Strength Condition. Res. 28, 308–318. 10.1519/JSC.0b013e31829b56b1 24476742

[B54] Meylan C. Cronin J. Oliver J. Hughes M. (2010). Reviews: talent identification in soccer: the role of maturity status on physical, physiological and technical characteristics. Int. J. Sports Sci. Coach. 5, 571–592. 10.1260/1747-9541.5.4.571

[B55] Mills A. Butt J. Maynard I. Harwood C. (2012). Identifying factors perceived to influence the development of elite youth football academy players. J. Sports Sci. 30, 1593–1604. 10.1080/02640414.2012.710753 22888797

[B56] Mirwald R. Baxter-Jones A. Bailey D. Beunen G. (2002). An assessment of maturity from anthropometric measurements. Med. Sci. Sports Exerc. 34, 689–694. 10.1249/00005768-200204000-00020 11932580

[B57] Murr D. Feichtinger P. Larkin P. O ‘Connor D. Höner O. (2018a). Psychological talent predictors in youth soccer: a systematic review of the prognostic relevance of psychomotor, perceptual-cognitive and personality-related factors. PLoS ONE 13:e0205337. 10.1371/journal.pone.0205337 30321221 PMC6188900

[B58] Murr D. Larkin P. Höner O. (2021). Decision-making skills of high-performance youth soccer players. German J. Exerc. Sport Res. 51, 102–111. 10.1007/s12662-020-00687-2

[B59] Murr D. Raabe J. Höner O. (2018b). The prognostic value of physiological and physical characteristics in youth soccer: a systematic review. Euro. J. Sport Sci. 18, 62–74. 10.1080/17461391.2017.1386719 29161984

[B60] Musculus L. Lobinger B. (2018). Psychological characteristics in talented soccer players – recommendations on how to improve coaches' assessment. Front. Psychol. 9:41. 10.3389/fpsyg.2018.00041 29459839 PMC5807374

[B61] Myburgh G. Cumming S. Malina R. (2019). Cross-sectional analysis investigating the concordance of maturity status classifications in elite caucasian youth tennis players. Sports Med. Open 5:27. 10.1186/s40798-019-0198-8 31264052 PMC6603099

[B62] Nortje L. Dicks M. Coopoo Y. Savelsbergh G. (2014). Put your money where your mouth is: verbal self-reported tactical skills versus on-line tactical performance in soccer. Int. J. Sports Sci. Coach. 9, 321–334. 10.1260/1747-9541.9.2.32133936729

[B63] O'Connor D. Larkin P. Williams A. (2016). Talent identification and selection in elite youth football: an Australian context. Euro. J. Sport Sci. 16, 837–844. 10.1080/17461391.2016.1151945 26923813

[B64] Roca A. Williams A. Ford P. (2012). Developmental activities and the acquisition of superior anticipation and decision making in soccer players. J. Sports Sci. 30, 1643–1652. 10.1080/02640414.2012.701761 22769067

[B65] Romann M. Fuchslocher J. (2013). Relative age effects in Swiss junior soccer and their relationship with playing position. Euro. J. Sport Sci. 13, 356–363. 10.1080/17461391.2011.635699 23834540

[B66] Sarmento H. Anguera M. Pereira A. Araujo D. (2018). Talent identification and development in male football: a systematic review. Sports Med. 48, 907–931. 10.1007/s40279-017-0851-7 29299878

[B67] Saward C. Hulse M. Morris J. Goto H. Sunderland C. Nevill M. (2020). Longitudinal physical development of future professional male soccer players: implications for talent identification and development? Front. Sports Active Living. 2:578203. 10.3389/fspor.2020.578203 33345142 PMC7739714

[B68] Sheppard J. Dawes J. Jeffreys I. Spiteri T. Nimphius S. (2014). Broadening the view of agility: a scientific review of the literature. J. Austral. Strength Condition. 22, 6–25.

[B69] Sheppard J. Young W. (2006). Agility literature review: classifications, training and testing. J. Sports Sci. 24, 919–932. 10.1080/02640410500457109 16882626

[B70] Sieghartsleitner R. Zuber C. Zibung M. Charbonnet B. Conzelmann A. (2019b). Talent selection in youth football: specific rather than general motor performance predicts future player status of football talents. Curr. Issues Sport Sci. 10.15203/CISS_2019.01130787649

[B71] Sieghartsleitner R. Zuber C. Zibung M. Conzelmann A. (2019a). Science or coaches' eye?–both! Beneficial collaboration of multidimensional measurements and coach assessments for efficient talent selection in elite youth football. J. Sports Sci. Med. 18, 32–43. PMID: 30787649 30787649 PMC6370964

[B72] Suppiah H. T. Low C. Y. Chia M. (2015). Detecting and developing youth athlete potential: different strokes for different folks are warranted. Br. J. Sports Med. 49, 878–882. 10.1136/bjsports-2015-094648 25907182

[B73] Toering T. Elferink-Gemser M. Jordet G. Visscher C. (2009). Self-regulation and performance level of elite and non-elite youth soccer players. J. Sports Sci. 27, 1509–1517. 10.1080/02640410903369919 19967593

[B74] Travassos B. Araujo D. Davids K. O'hara K. Leitão J. Cortinhas A. (2013). Expertise effects on decision-making in sport are constrained by requisite response behaviours–a meta-analysis. Psychol. Sport Exerc. 14, 211–219. 10.1016/j.psychsport.2012.11.002

[B75] Unnithan V. White J. Georgiou A. Iga J. Drust B. (2012). Talent identification in youth soccer. J. Sports Sci. 30, 1719–1726. 10.1080/02640414.2012.731515 23046427

[B76] Van Yperen N. (2009). Why some make it and others do not: identifying psychological factors that predict career success in professional adult soccer. Sport Psychol. 23, 317–329. 10.1123/tsp.23.3.317

[B77] Votteler A. Höner O. (2017). Cross-sectional and longitudinal analyses of the relative age effect in German youth football. German J. Exerc. Sport Res. 47, 194–204. 10.1007/s12662-017-0457-0

[B78] Ward P. Ericsson K. Williams A. (2013). Complex perceptual-cognitive expertise in a simulated task environment. J. Cogn. Eng. Decision Making 7, 231–254. 10.1177/1555343412461254

[B79] Williams A. Ford P. Drust B. (2020). Talent identification and development in soccer since the millennium. J. Sports Sci. 38, 1199–1210. 10.1080/02640414.2020.1766647 32568000

[B80] Williams A. Reilly T. (2000). Talent identification and development in soccer. J. Sports Sci. 18, 657–667. 10.1080/02640410050120041 11043892

[B81] Young W. Rayner R. Talpey S. (2021). It's time to change direction on agility research: a call to action. Sports Med. Open 7:12. 10.1186/s40798-021-00304-y 33580424 PMC7881072

