# Supplementary material for: Nationwide Subjective and Objective Assessments of Potential Talent Predictors in Elite Youth Soccer: An Investigation of Prognostic Validity in a Prospective Study
Source: Front Sports Act Living. 2021 May 28;3:638227. doi: 10.3389/fspor.2021.638227 (PMC8193982; doi:10.3389/fspor.2021.638227)
Supplement: Supplementary file 1 [file Table_1.docx]

**Table S1a.** Players’ kicking skills: Key points and their explanations for coaches’ ratings

| **Items** | **Key points** | **Explanation of the key points** |
| --- | --- | --- |
| 1. *Kicking techniques with the dominant foot* | - Variability - Precision - Speed | Competence center players can …   - use various kicking techniques: inside, top, outside of the foot. - pass/shoot balls with high variability: e.g. straight balls (high and flat), curve balls. - pass/shoot with high precision. - pass/shoot balls at a speed that is appropriate for the situation: e.g. firm passes, but also ability to differentiate when playing. - successfully/effectively pass/shoot balls from different situations: e.g. without/with opponent pressure, without/with time pressure. |
| 1. *Kicking techniques with the non-dominant foot* | - Variability - Precision - Speed | Competence center players can …   - use various kicking techniques: inside, top, outside of the foot. - pass/shoot balls with high variability: e.g. straight balls (high and flat), curve balls. - pass/shoot with high precision. - pass/shoot balls at a speed that is appropriate for the situation: e.g. firm passes, but also ability to differentiate when playing. - successfully/effectively pass/shoot balls from different situations: e.g. without/with opponent pressure, without/with time pressure. |
| 1. *Heading* | - Variability - Precision - Timing | Competence center players can …   - use various heading techniques: e.g. from standing, from a one-legged/two-legged jump. - head precisely and effectively: e.g. targeted. - time their jump to meet the ball: e.g. springing action, meeting at the highest point. |
