# Supplementary material for: Nationwide Subjective and Objective Assessments of Potential Talent Predictors in Elite Youth Soccer: An Investigation of Prognostic Validity in a Prospective Study
Source: Front Sports Act Living. 2021 May 28;3:638227. doi: 10.3389/fspor.2021.638227 (PMC8193982; doi:10.3389/fspor.2021.638227)
Supplement: Supplementary file 2 [file Table_2.docx]

**Table S1b.** Players’ endurance ability: Key points and their explanations for coaches’ ratings

| **Item** | **Key points** | **Explanation of the key points** |
| --- | --- | --- |
| 1. *Endurance* | - Physical stamina | Competence center players …   - not fade physically or psychologically during the entire duration of training sessions or competitions: e.g. even at the end of the training session/game, players can still perform high-intensity movement sequences of high quality, quickly shrug off intensive strain (no or only slight physical fatigue becomes apparent), still make the right decisions (quickly), and the ability to absorb information and willingness to run remain high. |
