# Supplementary material for: Nationwide Subjective and Objective Assessments of Potential Talent Predictors in Elite Youth Soccer: An Investigation of Prognostic Validity in a Prospective Study
Source: Front Sports Act Living. 2021 May 28;3:638227. doi: 10.3389/fspor.2021.638227 (PMC8193982; doi:10.3389/fspor.2021.638227)
Supplement: Supplementary file 3 [file Table_3.docx]

**Table S1c.** Players’ individual tactical skills: Key points and their explanations for coaches’ ratings

| **Items** | **Key points** | **Explanation of the key points** |
| --- | --- | --- |
| 1. *Behavior in offensive situations before ball-related actions* | - Pre-orientation - Offering/Creating Space | Competence center players can …   - orientate themselves in such a way that they make an appropriate decision: e.g. find an open position, look over their shoulders. - make themselves available in such a way that they are playable or create space in which another player becomes playable: e.g. separate themselves from a defender. |
| 1. *Behavior in offensive situations during ball-related actions* | - First touch - Orientation on the ball - Situation-appropriate decision-making | Competence center players can …   - play the ball with the first touch according to the situation: e.g. in the new direction of play, in the open space/away from the opponent, secure the ball. - orient themselves while they are on the ball: e.g. glance away from the ball, view the next situation. - decide appropriately: e.g. pass the ball to a teammate, dribble with the ball, shoot the ball at the goal. |
| 1. *Behavior in offensive situations after ball-related actions* | - Re-orientation - Follow-up action | Competence center players can …   - act according to a new situation after a ball-related action: e.g. pass the ball and immediately offer again, be offset to back up teammates, be playable again, create space for teammates. |
| 1. *Behavior in defensive situations before ball-related actions* | - Preparing for defensive action | Competence center players can …   - correctly assess which game situation is coming to them and prepare the defensive action accordingly: e.g. pay attention to the positional game, shorten the distance to the opponent, pay attention to the body position of the opponent, anticipate the pass. - act correctly according to the situation when expecting the ball: e.g. stand within range so that the pass can be intercepted and the opponent can be attacked. |
| 1. *Behavior in defensive situations during ball-related actions* | - Distance - Body position - Being in the passing lane - Pressure | Competence center players can …   - act correctly with dribbling opponents: e.g., shorten the distance to the opponent, then direct him/her sideways into "basketball position," pay attention to the opponents’ body position, foot orientation and the direction of rotation. - make the right decision with opponents who stand with their backs turned: e.g. put the opponents under pressure and do not let them turn. - position themselves correctly: e.g. trying to get into passing lane to the opponent. |
| 1. *Behavior in defensive situations after ball-related actions* | - Follow-up action | Competence center players can …   - act appropriately after winning the ball: e.g. secure possession or switch directly to the attack, become playable quickly. - act correctly after making a mistake e.g. immediately pursue the ball. |
| 1. *Game intelligence* | - Transitional game - Overall game awareness - Tactical group behavior - Speed of action | Competence center players can …   - show a situation-appropriate transitional awareness after winning the ball: e.g. secure possession, pass in the depth quickly, become playable by quickly running into space, transition quickly. - show a situation-appropriate transitional awareness after losing the ball: e.g. immediately trigger counter-pressing, drop, transition quickly. - behave correctly in the tactical group context: e.g. always be playable on offense, create space, move forward, change positions with teammates, slot into the defense’s weak side, help teammates through double-teams. - show a good game awareness: e.g. play purposefully. - quickly decide on game actions that are most likely to succeed: e.g. anticipation (thinking about the next action). - find good solutions for tight situations. |
