# Supplementary material for: Nationwide Subjective and Objective Assessments of Potential Talent Predictors in Elite Youth Soccer: An Investigation of Prognostic Validity in a Prospective Study
Source: Front Sports Act Living. 2021 May 28;3:638227. doi: 10.3389/fspor.2021.638227 (PMC8193982; doi:10.3389/fspor.2021.638227)
Supplement: Supplementary file 4 [file Table_4.docx]

**Table S1d.** Players’ psychosocial skills: Key points and their explanations for coaches’ ratings

| **Items** | **Key points** | **Explanation of the key points** |
| --- | --- | --- |
| 1. *Motivational skills* | - Goal setting - Own responsibility - Willingness to learn | Competence center players can …   - set themselves helpful goals, e.g.:   - know their athletic goals (for the next training or game, for the coming months).   - set demanding but realistic goals.   - talk about what they want to achieve ("I want to improve personally"), rather than what they want to avoid ("I don't want to make so many mistakes").   - are happy about personal achievements (regardless of whether others are better or worse). - act independently, e.g.:   - are reliable and prepared to practice even without direct supervision (e.g. for training drills in small groups, independent exercises for core stability at home).   - take on tasks even without prompting (equipment preparation, tidying up the pitch, tidying the dressing room, etc.). - learn with joy and under their own initiative, e.g.:   - like new or difficult tasks in training.   - ask for feedback and what they can improve further.   - are on the pitch to practice, outside of training or without a coach. |
| 1. *Volitional skills* | - Focusing/Self-control - Effort readiness/goal tracking - Self-assessment | Competence center players …   - can focus and control oneself, e.g.:   - talk about or think about their task (they don't think about results, mistakes, other players, spectators) during trainings or game preparation.   - show little reaction to distraction (calls, provocations).   - react in a controlled manner to difficult situations (own mistakes, incorrect decisions by coaches or referees).   - have positive body language even in difficult situations (e.g. upright posture and head up).   - have adequate body tension (tension during practice or play, relaxation during breaks). - can pursue their goals (even in the face of adversity), e.g.:   - push their performance limits in training or play.   - show a positive reaction immediately after failure or mistakes in training or games   - work (voluntarily) on the necessary facets to achieve their goals (individual training, sacrificing other leisure activities). - have adequate self-confidence, e.g.:   - can realistically estimate their performance on the pitch (not too high or too low).   - are convinced of their skills on the pitch, even in difficult situations.   - can accurately assess what they need to do to successfully complete a task.   - demand the ball in difficult situations. |
| 1. *Social skills* | - Team skills - Leadership | Competence center players can …   - integrate themselves into a team, e.g.:   - accept different opinions when making decisions or discussions.   - listen carefully and let others speak out (coaches, supervisors, teammates).   - accept team decisions or tasks (also at their expense).   - are interested in the condition of their teammates (e.g. injuries, private situations).   - offer support and assistance to others (on and off the pitch).   - express their opinion.   - can deal with criticism or feedback and benefit from it. - take the lead or be guided if necessary, e.g.:   - make decisions for themselves and others on and off the pitch.   - are respected by their teammates or asked for their opinion.   - coach teammates in a helpful manner during training or games.   - make clear and easy-to-understand announcements for their teammates.   - accept instructions or coaching from leaders or managers. |
