# Supplementary material for: Nationwide Subjective and Objective Assessments of Potential Talent Predictors in Elite Youth Soccer: An Investigation of Prognostic Validity in a Prospective Study
Source: Front Sports Act Living. 2021 May 28;3:638227. doi: 10.3389/fspor.2021.638227 (PMC8193982; doi:10.3389/fspor.2021.638227)
Supplement: Supplementary file 5 [file Table_5.docx]

**Table S2.** Odds ratios for being selected for a youth academy for players with high (i.e., *PR* > 66.67) vs. medium (33.33 < *PR* ≤ 66.67) or high vs. low (*PR* ≤ 33.33) test results regarding the subjectively (printed in italics) and objectively evaluated performance factors (separated by age class, U12 - U15, *N* = 13,869)

| **Performance factor ^(#)^** | **Performance group comparison** | **Odds ratio** | | | |
| --- | --- | --- | --- | --- | --- |
|  |  | **[95% CI]** | | | |
|  |  | **U12** | **U13** | **U14** | **U15 ^(+)^** |
| *Tactical skills* | High vs. medium | 2.66 [2.20; 3.23] | 2.46 [1.94; 3.11] | 2.39 [1.73; 3.30] | 2.11 [1.21; 3.67] |
|  | High vs. low | 6.91 [5.11; 9.35] | 6.43 [4.35; 9.51] | 4.17 [2.63; 6.61] | 16.67 [3.97; 69.90] |
| *Kicking skills* | High vs. medium | 1.90 [1.58; 2.29] | 2.61 [2.04; 3.33] | 1.92 [1.39; 2.64] | 3.33 [1.90; 5.82] |
|  | High vs. low | 6.58 [5.00; 8.65] | 5.07 [3.67; 6.99] | 4.84 [3.12; 7.49] | 19.19 [4.58; 80.49] |
| *Psych. skills* | High vs. medium | 2.04 [1.69; 2.46] | 1.96 [1.54; 2.50] | 1.38 [1.00; 1.90] | 1.07 [0.60; 1.92] |
|  | High vs. low | 3.26 [2.53; 4.20] | 3.38 [2.41; 4.74] | 2.40 [1.53; 3.77] | 3.16 [1.16; 8.62] |
| Sprint (20m) | High vs. medium | 2.10 [1.73; 2.55] | 2.23 [1.74; 2.86] | 2.02 [1.45; 2.80] | 1.29 [0.72; 2.33] |
|  | High vs. low | 4.87 [3.79; 6.26] | 5.81 [4.15; 8.13] | 5.61 [3.60; 8.75] | 2.61 [1.27; 5.38] |
| Agility (CODS) | High vs. medium | 1.73 [1.40; 2.09] | 1.31 [1.01; 1.69] | 1.02 [0.73; 1.41] | 1.20 [1.15; 2.16] |
|  | High vs. low | 2.25 [1.81; 2.79] | 1.77 [1.34; 2.32] | 1.97 [1.34; 2.91] | 2.84 [1.35; 5.96] |
| Dribbling | High vs. medium | 2.29 [1.87; 2.81] | 1.68 [1.30; 2.16] | 1.46 [1.05; 2.02] | 2.16 [1.15; 4.06] |
|  | High vs. low | 3.07 [2.46; 3.83] | 2.48 [1.87; 3.29] | 2.88 [1.94; 4.27] | 2.73 [1.38; 5.38] |
| Ball control | High vs. medium | 1.66 [1.37; 2.03] | 1.42 [1.10; 1.83] | 1.54 [1.09; 2.17] | 1.49 [0.83; 2.68] |
|  | High vs. low | 2.70 [2.16; 3.38] | 1.82 [1.38; 2.39] | 1.63 [1.15; 2.32] | 3.46 [1.62; 7.40] |
| Juggling | High vs. medium | 2.04 [1.59; 2.62] | 1.57 [1.16; 2.11] | 1.34 [0.95; 1.90] | 1.43 [0.78; 2.65] |
|  | High vs. low | 2.53 [2.09; 3.06] | 2.48 [1.93; 3.18] | 2.55 [1.78; 3.66] | 2.67 [1.36; 5.25] |

Note. (#): As the performance factor endurance was assessed by one single item on a 4-point scale, no meaningful categorizations for high, medium, and low performance according to the chosen percentile ranks could be calculated. Therefore, odds ratios were not computed for this predictor. (+): Due to the low sample sizes in U15 (particularly in the selected group), estimations for the (in parts rather high) odds ratios and the corresponding 95% confidence intervals might be biased and, therefore, must be considered with caution.
