# Supplementary material for: Nationwide Subjective and Objective Assessments of Potential Talent Predictors in Elite Youth Soccer: An Investigation of Prognostic Validity in a Prospective Study
Source: Front Sports Act Living. 2021 May 28;3:638227. doi: 10.3389/fspor.2021.638227 (PMC8193982; doi:10.3389/fspor.2021.638227)
Supplement: Supplementary file 6 [file Table_6.docx]

**Table S3a.** Logistic regression results for the prediction of players future success (selected for a YA 3 seasons later) in dependence of the subjective assessment (model 1, separated by age group)

| Age Group | Omnibus-Tests | | | Predictor | Logistic regression coefficients | | | | *(e^b^)^SD^*  _(#)_ |  |
| --- | --- | --- | --- | --- | --- | --- | --- | --- | --- | --- |
|  |  |  |  |  |  |  |  |  |  |  |
|  |  |  |  |  |  |  |  |  |  |  |
|  | χ^2^ *(df)* | *p* | Nagelkerke *R*^2^ |  | *b* | Wald | *p* | *e^b^* [95%-CI] |  |  |
| U12 | 305.20 (4) | < .001 | 0.10 | Constant | -4.39 | - | - | - | - |  |
|  |  |  |  | *Tactical Skills* | 1.14 | 61.17 | < .01 | 3.12 [2.34; 4.14] | 1.97 |  |
|  |  |  |  | *Kicking Skills* | 0.47 | 14.23 | < .01 | 1.6 [1.25; 2.05] | 1.31 |  |
|  |  |  |  | *Psychosocial Skills* | -0.34 | 10.18 | < .01 | 0.71 [0.58; 0.88] | 0.80 |  |
|  |  |  |  | *Endurance* | 0.04 | 0.17 | 0.68 | 1.04 [0.88; 1.22] | - |  |
| U13 | 192.20 (4) | < .001 | 0.11 | Constant | -4.49 | - | - | - | - |  |
|  |  |  |  | *Tactical Skills* | 0.97 | 29.91 | < .01 | 2.65 [1.87; 3.75] | 1.76 |  |
|  |  |  |  | *Kicking Skills* | 0.75 | 22.37 | < .01 | 2.12 [1.55; 2.89] | 1.52 |  |
|  |  |  |  | *Endurance* | -0.25 | 5.46 | 0.02 | 0.78 [0.63; 0.96] | 0.83 |  |
|  |  |  |  | *Psychosocial Skills* | -0.05 | 0.14 | 0.71 | 0.95 [0.73; 1.24] | - |  |
| U14 | 73.75 (4) | < .001 | 0.07 | Constant | -3.94 | - | - | - | - |  |
|  |  |  |  | *Tactical Skills* | 0.87 | 13.02 | < .01 | 2.39 [1.49; 3.84] | 1.66 |  |
|  |  |  |  | *Kicking Skills* | 0.55 | 6.85 | 0.01 | 1.73 [1.15; 2.61] | 1.36 |  |
|  |  |  |  | *Psychosocial Skills* | -0.33 | 3.76 | 0.05 | 0.72 [0.52; 1.00] | - |  |
|  |  |  |  | *Endurance* | -0.04 | 0.05 | 0.81 | 0.97 [0.72; 1.30] | - |  |
| U15 | 40.87 (4) | < .001 | 0.10 | Constant | -5.49 | - | - | - | - |  |
|  |  |  |  | *Tactical Skills* | 1.49 | 10.89 | < .01 | 4.45 [1.83; 10.81] | 2.32 |  |
|  |  |  |  | *Psychosocial Skills* | -0.45 | 2.34 | 0.13 | 0.64 [0.36; 1.14] | - |  |
|  |  |  |  | *Kicking Skills* | 0.55 | 2.01 | 0.16 | 1.73 [0.81; 3.67] | - |  |
|  |  |  |  | *Endurance* | -0.15 | 0.31 | 0.58 | 0.86 [0.51; 1.45] | - |  |

Note: Predicters were ordered by increasing values with regard to the Wald-statistic. (#) In order to facilitate comparisons for effect sizes of individual predictors, the odds ratio coefficients *e^b^* were additionally adjusted to the standard deviations of the respective age group (Höner & Votteler, 2016). The resulting *(e^b^)^SD^* represent the relative change of the likelihood for being selected for a YA by a one standard deviation increase within the considered predictor.
