# Supplementary material for: Nationwide Subjective and Objective Assessments of Potential Talent Predictors in Elite Youth Soccer: An Investigation of Prognostic Validity in a Prospective Study
Source: Front Sports Act Living. 2021 May 28;3:638227. doi: 10.3389/fspor.2021.638227 (PMC8193982; doi:10.3389/fspor.2021.638227)
Supplement: Supplementary file 7 [file Table_7.docx]

|  |  |
| --- | --- |

**Table S3b.** Logistic regression results for the prediction of players future success (selected for a YA 3 seasons later) in dependence of the objective assessment (model 2, separated by age group)

| Age Group | Omnibus-Tests | | | Predictor | Logistic regression coefficients | | | | *(e^b^)^SD^*  _(#)_ |  |
| --- | --- | --- | --- | --- | --- | --- | --- | --- | --- | --- |
|  |  |  |  |  |  |  |  |  |  |  |
|  |  |  |  |  |  |  |  |  |  |  |
|  | χ^2^ *(df)* | *p* | Nagelkerke *R*^2^ |  | *b* | Wald | *p* | *e^b^* [95%-CI] |  |  |
| U12 | 388.02 (5) | < .001 | 0.13 | Constant | 17.31 | - | - | - | - |  |
|  |  |  |  | Sprint | -3.92 | 144.38 | < .01 | 0.02 [0.01; 0.04] | 1.87 |  |
|  |  |  |  | Dribbling | -0.53 | 41.09 | < .01 | 0.59 [0.50; 0.69] | 1.49 |  |
|  |  |  |  | Juggling | 0.07 | 33.27 | < .01 | 1.07 [1.04; 1.09] | 1.23 |  |
|  |  |  |  | Ball control | -0.18 | 19.76 | < .01 | 0.84 [0.77; 0.90] | 1.27 |  |
|  |  |  |  | Agility (CODS) | 0.21 | 2.25 | 0.13 | 1.24 [0.94; 1.63] | - |  |
| U13 | 245.51 (5) | < .001 | 0.14 | Constant | 15.89 | - | - | - | - |  |
|  |  |  |  | Sprint | -4.68 | 136.85 | < .01 | 0.01 [0.00; 0.02] | 2.15 |  |
|  |  |  |  | Juggling | 0.07 | 35.24 | < .01 | 1.07 [1.05; 1.10] | 1.35 |  |
|  |  |  |  | Dribbling | -0.54 | 23.72 | < .01 | 0.58 [0.47; 0.72] | 1.45 |  |
|  |  |  |  | Agility (CODS) | 0.54 | 8.85 | < .01 | 1.71 [1.20; 2.45] | 0.81 |  |
|  |  |  |  | Ball control | -0.08 | 2.03 | 0.15 | 0.93 [0.83; 1.03] | - |  |
| U14 | 134.2 (5) | < .001 | 0.13 | Constant | 18.3 | - | - | - | - |  |
|  |  |  |  | Sprint | -4.66 | 81.56 | < .01 | 0.01 [0.00; 0.03] | 2.23 |  |
|  |  |  |  | Dribbling | -0.65 | 16.12 | < .01 | 0.52 [0.38; 0.72] | 1.52 |  |
|  |  |  |  | Juggling | 0.03 | 5.76 | 0.02 | 1.03 [1.01; 1.06] | 1.19 |  |
|  |  |  |  | Ball control | -0.13 | 2.98 | 0.08 | 0.88 [0.76; 1.02] | - |  |
|  |  |  |  | Agility (CODS) | 0.34 | 2.04 | 0.15 | 1.41 [0.88; 2.26] | - |  |
| U15 | 33.51 (5) | < .001 | 0.08 | Constant | 14.37 | 12.11 | < .01 | - | - |  |
|  |  |  |  | Sprint | -2.84 | 8.78 | < .01 | 0.06 [0.01; 0.38] | 1.55 |  |
|  |  |  |  | Dribbling | -0.74 | 6.99 | 0.01 | 0.48 [0.28; 0.83] | 1.71 |  |
|  |  |  |  | Juggling | 0.04 | 2.97 | 0.08 | 1.04 [1.00; 1.09] | - |  |
|  |  |  |  | Ball control | -0.25 | 2.91 | 0.09 | 0.78 [0.58; 1.04] | - |  |
|  |  |  |  | Agility (CODS) | 0.17 | 0.15 | 0.70 | 1.19 [0.49; 2.84] | - |  |

Note: Predicters were ordered by increasing values with regard to the Wald-statistic. (#) In order to facilitate comparisons for effect sizes of individual predictors, the odds ratio coefficients *e^b^* were additionally adjusted to the standard deviations of the respective age group (Höner & Votteler, 2016 The resulting *(e^b^)^SD^* represent the relative change of the likelihood for being selected for a YA by a one standard deviation increase within the considered predictor. For negatively coded predictors, the adjusted odds ratios were inverted and displayed as (*e^b^)^-SD^*.
